# Probing the limits of Q-tag bioconjugation of antibodies†
†Electronic supplementary information (ESI) available: Methods section including the synthesis of Ab-labelling reagents, Ab conjugation methods, chromatography and native MS. See DOI: 10.1039/c9cc02303h


**DOI:** 10.1039/c9cc02303h

**Published:** 2019-09-03

**Authors:** Cristina Marculescu, Abirami Lakshminarayanan, Joseph Gault, James C. Knight, Lisa K. Folkes, Thomas Spink, Carol V. Robinson, Katherine Vallis, Benjamin G. Davis, Bart Cornelissen

**Affiliations:** a CRUK/MRC Oxford Institute for Radiation Oncology , Department of Oncology, University of Oxford , Oxford , OX3 7DQ , UK . Email: kathrine.vallis@oncology.ox.ac.uk ; Email: bart.cornelissen@oncology.ox.ac.uk; b Chemistry Research Laboratory , University of Oxford , Oxford , OX1 3TA , UK . Email: ben.davis@chem.ox.ac.uk

## Abstract

Site-selective labelling of antibodies (Abs) can circumvent problems from heterogeneity of conventional conjugation. Here, we evaluate the industrially-applied chemoenzymatic ‘Q-tag’ strategy based on transglutaminase-mediated (TGase) amide-bond formation in the generation of ^89^Zr-radiolabelled antibody conjugates. We show that, despite previously suggested high regioselectivity of TGases, in the anti-Her2 Ab Herceptin™ more precise native MS indicates only 70–80% functionalization at the target site (Q298_H_), in competition with modification at other sites, such as Q3_H_ critically close to the CDR1 region.

## Abstract 2

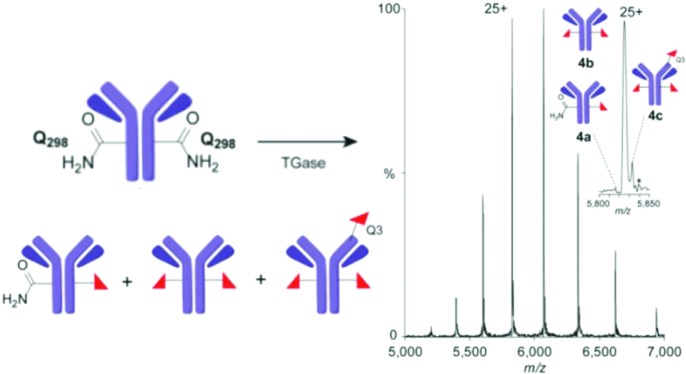
Precise analyses reveal that, while useful in reducing heterogeneity, the use of TGases in site-selective Ab modification may still create unwanted ‘off-site’ conjugates.

## 

Labelled antibodies (Abs) are vital clinical imaging tools and therapeutic agents.^1^ Generating conjugated Abs through site-specific conjugations that are more homogenously modified to clinically relevant standards is essential for future therapeutic use.^2^–^4^ Chemoenzymatic approaches can exploit the chemoselectivity and possible regioselectivity of even native residues in antibodies and can therefore enable ‘remodelling’ of existing antibodies.^5^–^8^ Transglutaminase (TGase) is one such enzyme that has been suggested to catalyze transamidation reactions of glutamine (Q) residues in a recognition sequence (the ‘Q-tag’) over other glutamines in heavy chains of IgGs, thus facilitating possible site-specific modification.^9^–^11^ As a consequence, TGase-mediated ‘Q-tag’ modification of Abs has been widely explored to generate Ab–drug conjugates,^12^,^13^ as well as labelled Abs,^9^,^10^,^12^,^14^ in both academia and industry.

Radiolabelled Abs find use in diagnostic imaging *via e.g.* Positron Emission Tomography (PET) or Single Photon Emission Computed Tomography (SPECT) as well as enabling great progress in immunotherapy.^15^ Zirconium-89 in particular has emerged as a powerful isotope for such applications. Its favorable half-life (∼3.3 days) is compatible with the slow clearance rate of Abs *in vivo*, allowing longer imaging whilst also providing high PET resolution.^16^^89^Zr-labelled Abs therefore represent a key demonstration system. Typically, ^89^Zr-labelled Abs are generated by initial conjugation of a suitable metal-chelator (*e.g.* siderophore deferoxamine (DFO)^17^) followed by radio-metal chelation.^18^ With few exceptions^19^–^21^ attachment of a metal ion chelator to Ab has been achieved by targeting nucleophilic ε-amines of several lysine (Lys) residues (Fig. 1A),^22^ resulting in heterogeneity.

**Fig. 1:**
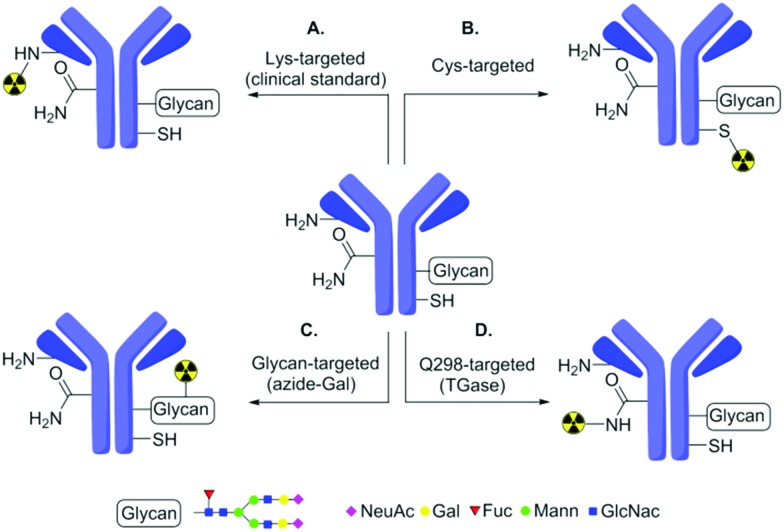
Strategies for ^89^Zr-radiolabelling of Abs. Traditional modification methods based on Lys (A) typically generate heterogeneity. To reduce heterogeneity, these have been extended by methods based on Cys (B), glycans (C) or glutamine targeting (D) using chemical or chemoenzymatic methods. (D) The ‘Q-tag’ system explored in this work has been previously proposed to be exclusively selective for Q298_H_ in antibodies.

To improve homogeneity, protein engineering can be combined with chemical modification to install and more-selectively label additional cysteine (Cys) residues (Fig. 1B).^19^ Alternatively, chemoenzymatic approaches can also be used to modify glycan residues on Ab (Fig. 1C). Whilst these can reduce heterogeneity compared to traditional methods, they may still yield partial heterogeneity due to, *e.g.* mixed glycosylation patterns or incomplete loading. Here, we show that an alternative, industrially-applied, chemoenzymatic method – the ‘Q-tag’ system (Fig. 1D)^23^ – allows successful generation of ^89^Zr-labelled Abs. Notably, whilst this improves homogeneity, our study also reveals previously unappreciated limits of Q-tag site-selectivity at sites likely to directly impair function.

The transamidation activity of TGases, which naturally cross-link Gln and Lys side-chains^23^ has been exploited previously to modify several proteins^24^ including in generation of Ab–drug (and other) conjugates.^25^–^27^ This method relies on a presumed high, but in fact rarely fully-characterized selectivity for certain peptide sequences containing Gln (so-called ‘Q-tags’). Amongst these is the sequence PWEEQYNST^11^ in IgG Abs containing a target Q298_H_ residue (Herceptin numbering), found close to the *N*-glycosylated N300_H_ (Fig. 1D). As a consequence of the glycosylation at site N300, site Q298 is typically sterically-occluded but can be revealed by prior treatment with the amidase PNGase, which converts glycosylated-N300 to D300 (Fig. 2).^28^

**Fig. 2:**
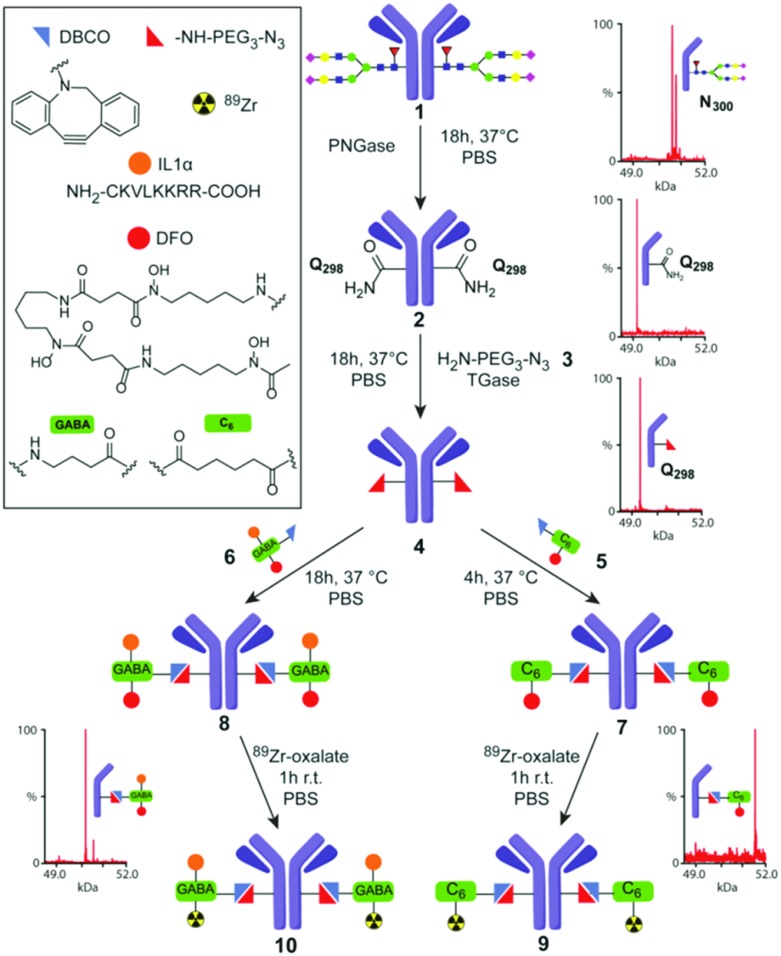
TGase-mediated, chemoenzymatic generation of ^89^Zr–Herceptin conjugates. A modular strategy based on ‘Q-tag’ allowed incorporation of variable moieties (see box). Sequential, chemoenzymatic remodeling using PNGase and TGase and then chemical conjugation was directly monitored by corresponding MS of heavy chain obtained under reducing, denaturing conditions (rLCMS) prior to final ^89^Zr chelation (see also ESI,† Scheme S1).

We applied a combined PNGase/TGase modification method to generate ^89^Zr-labelled Abs with reduced heterogeneity in the classical anti-Her2 Herceptin™ system. We designed a modular process that would allow near-direct comparison with prior results,^29^ through attachment of a DFO chelator to allow radiolabelling with ^89^Zr. This also enabled additional modification with other functional moieties (Fig. 2). To avoid metal-mediated conjugation strategies, which might inhibit/interfere with DFO chelation, we chose strain-promoted triazole formation^30^ for conjugation with a PEG–azidoamine^10^ as primary-amine co-substrate for TGase (Fig. 2).

The protein substrate, deglycosylated (dg) Herceptin (dg-Her, **2**), was generated by treating wild-type (wt) Herceptin (wt-Her, **1**) with PNGaseF,^31^ creating **2** as a D300_H_ Asp-variant of Her. Nearby and now accessible Q298_H_ of dg-Her **2** was then conjugated to the azidoamine H_2_N–CH_2_CH_2_–(OCH_2_CH_2_)_2_–N_3_ (**3**) using the TGase from *Streptomyces mobaraensis* to install an azide residue into the side chain of Q298_H_ (creating azido-Her **4**) for subsequent reaction with strained alkynes. Initial LCMS under reducing, denaturing conditions (rLCMS) and reducing SDS-PAGE analysis (ESI,† Table S1, method A), suggested that deglycosylation and azide-incorporation steps proceeded to completion, converting wt-Her **1** into desired products dg-Her **2** and then azido-Her **4** (Fig. 2). Retained reactivity of the azide moiety in azido-dg-Her **4** was confirmed using a Cy3-dye-containing alkyne (ESI,† Fig S1). Notably, no modification of the light chain was observed using these analytical methods (Fig. 3B). Together these traditional modes of analysis proved consistent with highly site-selective alterations guided by the Q-tag sequence, as previously proposed.

**Fig. 3:**
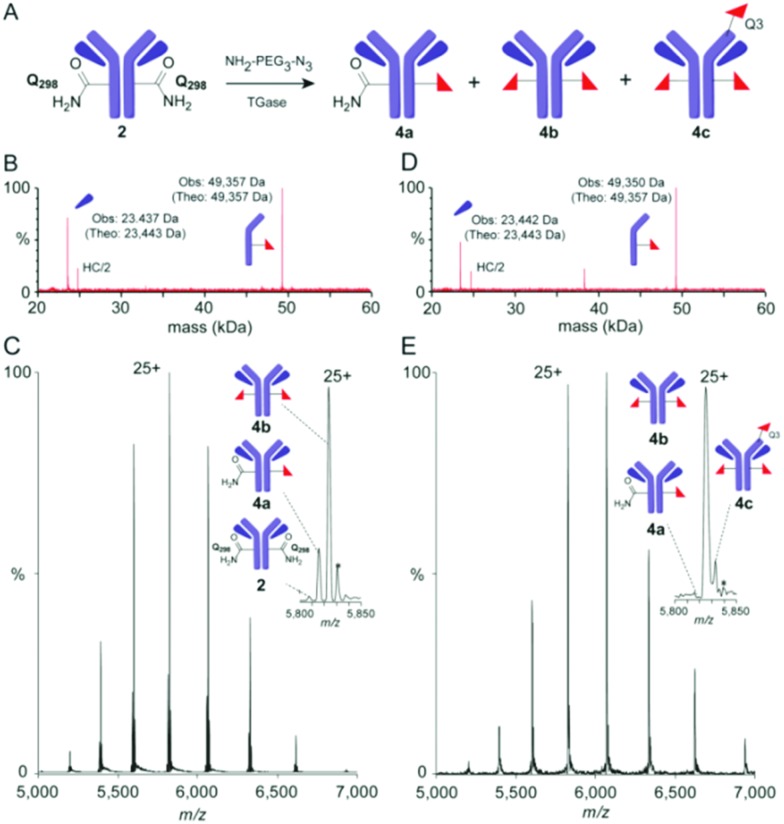
Precise monitoring of ‘Q-tag’ method reveals unexpected heterogeneity. (A) Reaction for TGase-mediated azide incorporation; (B) rLCMS and (C) nMS (spectrum and zoom into +25 charge state) of mixed azide-dg-Her 4 obtained using method A; (D) rLCMS and (E) nMS (spectrum and zoom of +25 charge state) of mixed azido-dg-Her **4** using method C reveals contaminant **4c** bearing modification at Q3_H_. Note: nMS (C and E) also show additional species (*)^33^ assigned to sequence variations (+176 Da), consistent with prior analyses.^6^

Prior work by us and others^6^,^32^ has demonstrated that the heteromultimeric nature of monoclonal Abs can lead to misleading quantitative analyses *via* rLCMS and that high resolution native MS (nMS) of intact monoclonal antibody conjugates can provide more precision and accuracy. nMS of dg-Her **2** confirmed complete removal of *N*-glycans of wt-Her **1** (ESI,† Fig S2). However, analyses of azido-dg-Her **4** generated under various conditions (ESI,† Table S1)^25^ unexpectedly revealed mixtures of different Ab species with varied conjugation states (Fig. 3: **4a–c**, azido copy numbers *a* = 1, *b* = 2, *c* = 3 plus unreacted **2**). Together, these data revealed azido-dg-Her **4**, formed under these conditions, is not homogeneous (Fig. 3C and E, also full MS data in ESI†).

Next, peptide mapping (tryptic-MS/MS) of azido-dg-Her **4** was used to dissect this surprising heterogeneity. This confirmed primary incorporation of modified residue azido-EG_3_-Q298_H_ at target site Q298_H_ (ESI,† Fig S3) could be achieved at levels of up to 70% (70.2% relative to **4b**). Notably, however, it also revealed that attempts to drive further conversions (*e.g.* method C, higher concentrations of Ab, amine, TGase) instead gave triply-modified product **4c**, bearing three azido moieties, at levels up to 14% (Fig. 3). Use of reduced equivalents of **3** gave only poor conversions (ESI,† Table S1, method E).

This was particularly surprising given the previously suggested selectivity^10^ of TGase for Q298_H_ and for the ‘Q-tag’ sequence. Tryptic-MS/MS analysis of azido-Her **4** generated using method C allowed unambiguous identification of Q3_H_ close to N-terminal CDR1 epitope-binding region as a third conjugation site (ESI,† Fig S4). Not only did this reveal limits of TGase-mediated ‘Q-tag’ conjugation, it also highlighted that the side-products contain modifications that may directly interfere with epitope binding due to proximity to CDRs.

Despite these unexpected and previously unappreciated limitations in the ‘Q-tag’, we were nonetheless able to generate useful target product mixtures (**4**) that were more homogeneous (∼80%) than those observed from typical chemical conjugations (*e.g. via* Lys – see ESI,† Fig. S7 for typical). This, in turn, allowed attachment of mono- and bi-functional moieties containing chelate DFO (using **5**), or DFO + peptide (using **6**). These modular DFO–alkyne **5** and DFO + IL1α-alkyne **6** reagents were themselves constructed using HATU-mediated amide bond formation (and maleimide conjugation in **6** – see ESI,† Scheme S1). Bi-functional **6** additionally contains a cell-penetrating peptide and nuclear localization sequence derived from hIL1α^34^ to test the introduction of a model peptide module that could allow interrogation of nuclear biomarkers in the future.^35^,^36^ These constructs and conjugations also allowed us to test the modularity of the TGase-based approach for building multi-functional Ab systems, by straightforward alteration of the corresponding alkyne-containing reaction partners. Reaction of **4** with both **5** or **6** proceeded with essentially full conversion (>95%), as judged by SDS-PAGE and rLCMS analysis (see ESI†), to yield conjugates [DFO]_2_-dg-Her **7** and [DFO + IL1α]_2_-dg-Her **8**, respectively, with near-identical copy number distribution ∼2 (Fig. 2). This copy number distribution was also confirmed by nMS analysis with fully conjugated products as major species. Slight peak broadening due to adventitious DFO-metal binding reduced quantification precision by nMS, (ESI,† Fig. S5 and S6). Analysis of conjugate stability over prolonged periods suggested good stability for **7**, but slow degradation of **8** (>6 months, *via* maleimide retro-Michael).

Radiolabelling of **7** and **8***via* chelation with ^89^Zr,^37^ yielded ^89^Zr-labelled dg-Her variants ^89^Zr·[DFO]_2_-dg-Her **9** and ^89^Zr·[DFO + IL1α]_2_-dg-Her **10** with radiochemical yields (RCY) of 94 ± 5% (*n* = 5) and 96 ± 5% (*n* = 7), respectively (Fig. 2). To allow side-by-side comparison with ^89^Zr–Herceptin conjugates obtained through conventional, random Lys-directed modification^38^,^39^ we also generated^37^ [DFO]mix-Her **11** (ESI,† Fig. S7). In contrast to site-selectively DFO-modified **8** and **9**, rLCMS analysis of [DFO]mix-Her **11** indicated high heterogeneity in both heavy and light chains (ESI,† Fig. S7). Radiolabelling of **11** with ^89^Zr provided ^89^Zr·[DFO]mix-Her **12** in RCY up to 98%.^37^

Retained biological functions of these Herceptin™ conjugates **7**, **8**, **11** were evaluated through determination of *in vitro* binding affinities (*K*_D_) to Her2 using a saturation-binding assay (ESI,† Fig. S8) and were not significantly different (*P* < 0.05) from wt Herceptin™. Importantly, ^89^Zr·[DFO + IL1α]_2_-dg-Her **10** proved highly stable in human serum at 37 °C, retaining radiolabel even after 4 days of incubation (ESI,† Fig. S9) and suggesting promising suitability for future *in vivo* use.

We have shown ‘Q-tag’ TGase-mediated Ab-conjugation yields less homogeneous conjugates than previously thought. This, the first precise analyses of intact TGase-generated Ab-conjugates conducted with nMS, reveals limitations in selectivity of widely-applied TGase. In the case of Herceptin, side products were formed with unwanted modification at sites critically close to CDRs. In preliminary experiments with murine anti-γH2AX antibody, (ESI,† Fig. S10), these limits of regioselectivity in the ‘Q-tag’-TGase method appear to be similar or worse. Our results were obtained with single amine **3** deemed efficient in prior studies;^11^ other amines may display altered selectivity. Indeed, unexpected TGase-driven modification of human proteins with endogenous amines has recently been noted,^40^ further highlighting the implications of TGase plasticity with respect to amine and protein substrates.

Not withstanding these limitations, the method does allow the creation of variants with improved homogeneity (∼80%) over traditional bioconjugations and enables a modular approach, described here, with potential for adding multiple functionalities in chelating moiety without any apparent gross effect on function. Surprisingly, prior *in vivo* comparisons^20^ have suggested that there are no differences between random attachment methods and more selective methods; future work will probe *in vivo* benefits of reduced heterogeneity.

The authors acknowledge support from CRUK (C5255/A15935), MRC (MC_PC_12004), and CRUK/EPSRC Cancer Imaging Centre Oxford (C5255/A16466). C. V. R. is supported by Wellcome Trust Investigator Award (104633/Z/14/Z), ERC Advanced Grant (641317) and MRC programme grant (MR/N020413/1). A. L. acknowledges funding by CRUK (CRUKDF 0318-AL). J. G. is a Junior Research Fellow at The Queen's College. We thank M. Mosley for assistance.

## Conflicts of interest

There are no conflicts of interest to declare.

## Supplementary Material

Supplementary information
