# Supplementary material for: Probing the limits of Q-tag bioconjugation of antibodies
Source: Chem Commun (Camb). 2019 Sep 3;55(76):11342–5. doi: 10.1039/c9cc02303h (PMC6788405; doi:10.1039/c9cc02303h)
Supplement: Supplementary file 1 [file CC-055-C9CC02303H-s001.pdf]

Electronic Supplementary Material (ESI) for ChemComm.
This journal is © The Royal Society of Chemistry 2019

Supplementary Information

for

Probing the limits of Q-tag bioconjugation of antibodies

Contents

1. Supplementary Table ………………………………………………………………………………… 3
2. Supplementary Scheme ……………………………………………………………………………… 4
3. Supplementary Figures ……………………………………………………………………………… 5
4. Materials and General Methods …………………………………………………………………… 16
5. Synthesis of antibody labelling reagents ……………………………………………………… 16
6. Chromatography Methods……………………………………………………………………………… 25
7. Antibody Conjugation Methods …………………………………………………………………….. 25
8. Native Mass Spectrometry Methods ……………………………………………………………… 33
9. MS/MS Methods ………………………………………………………………………………………… 40
10. Antibody Radioloading Methods ………………………………………………………………… 41
11. *In vitro* binding assay …………………………………………………………………………….. 41
12. Abbreviations ……………………………………………………………………………………………… 42
13. References ………………………………………………………………………………………………… 42

1. Supplementary Tables

Table S1. Various conditions screened for the synthesis of azido-dg-Her 4

Method	dg-Her 2 (mg/mL)	N_3-PEG_3-NH_2 3 (eq.)	TGase (U)/mg 2	Reaction time (h)
A	1	80	6	18
B	4	80	6	22
C	4.4	80 + 20 (after 20 h)	6 + 1.5 (after 20 h)	25
D	2	80 + 13 (after 19 h)	6 + 1 (after 19 h)	24
E	4.4	40	6	18

2. Supplementary Schemes

Scheme S1. Synthesis of DFO-alkyne 5 (A) and Dfo+Il1α-alkyne 6 (B).

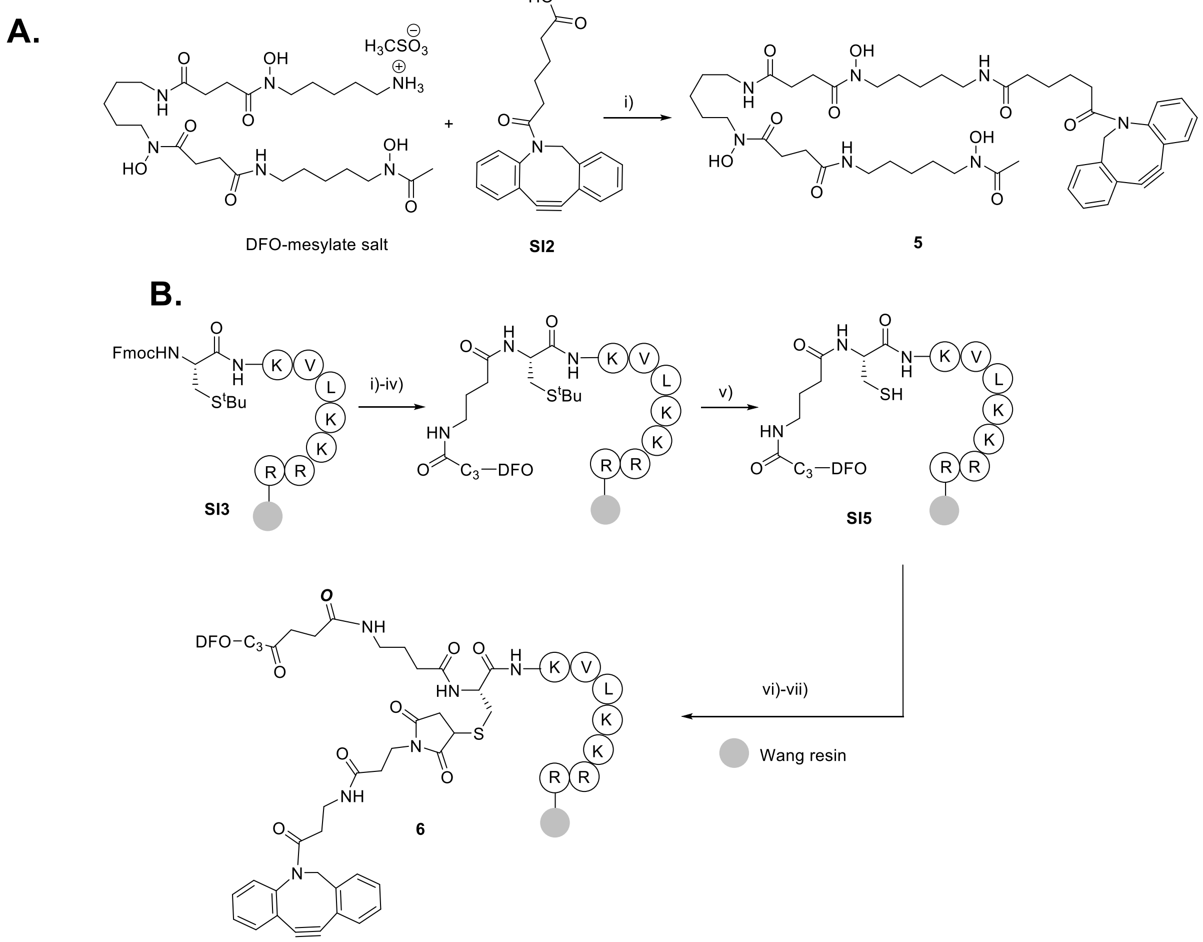

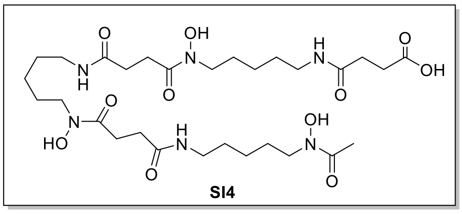

Reagents and conditions:
A. i) HATU, DIPEA, NMM, DMF, 48 h, r.t.
B. i) 20% Piperidine in DMF, 45 min, r.t.;
ii) 2 x (5 eq. FMOC-GABA, 5 eq. DIPEA, 5 eq. HATU), 1.5 h, r.t.;
iii) 20% Piperidine in DMF, 45 min, r.t.;
iv) 2 x (5 eq. DFO-acid **SI4**), 5 eq. DIPEA, 5 eq. HATU), 1.5 h, r.t.;
v) TFA : TIS : EDT = 9 5 : 2.5 : 2.5 (v/v);
vi) 1.2 eq. TCEP, PBS + 1 mM EDTA, 1 h, r.t.;
vii) 3 eq. Dibenzocyclooctyne-maleimide, PBS + 1 mM EDTA, 1 h, r.t..

3. Supplementary Figures

Figure S1. Structure of DBCO-Cy3 S1 and its conjugation to azido-dg-Her 4.

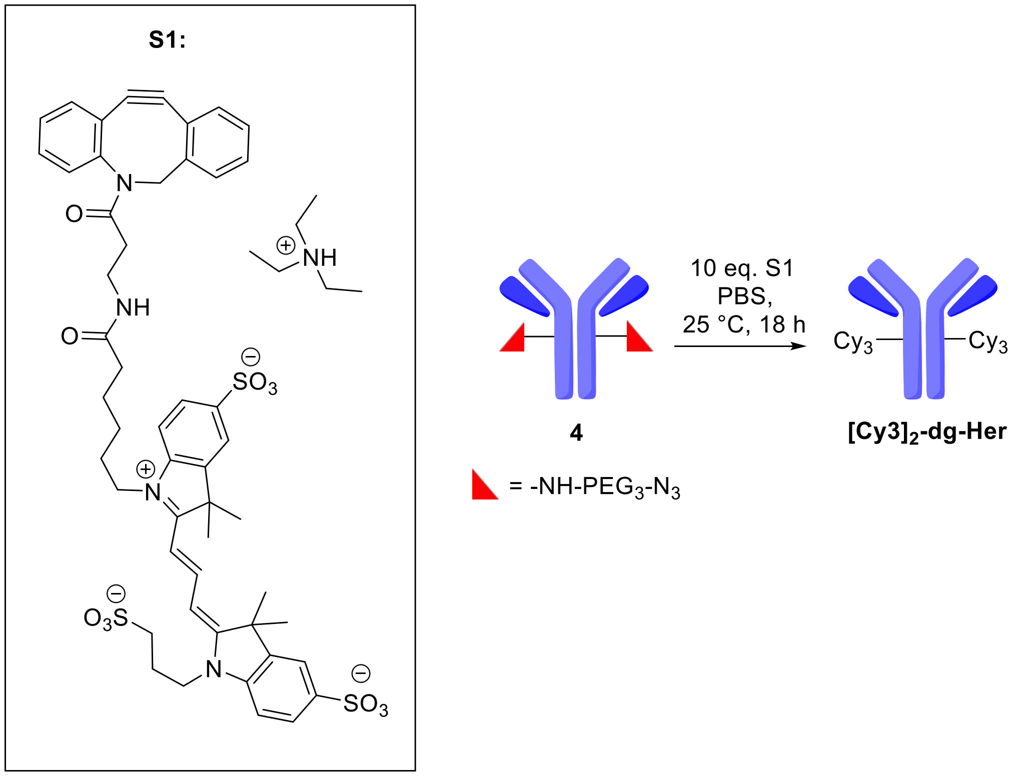

Figure S2. nMS analysis of dg-Her 2. The sample contains an additional species (*) assigned[1] to primary sequence variations (+ 176 Da, 12.3% abundance), consistent with the analysis performed by Parsons *et al.*.[2]

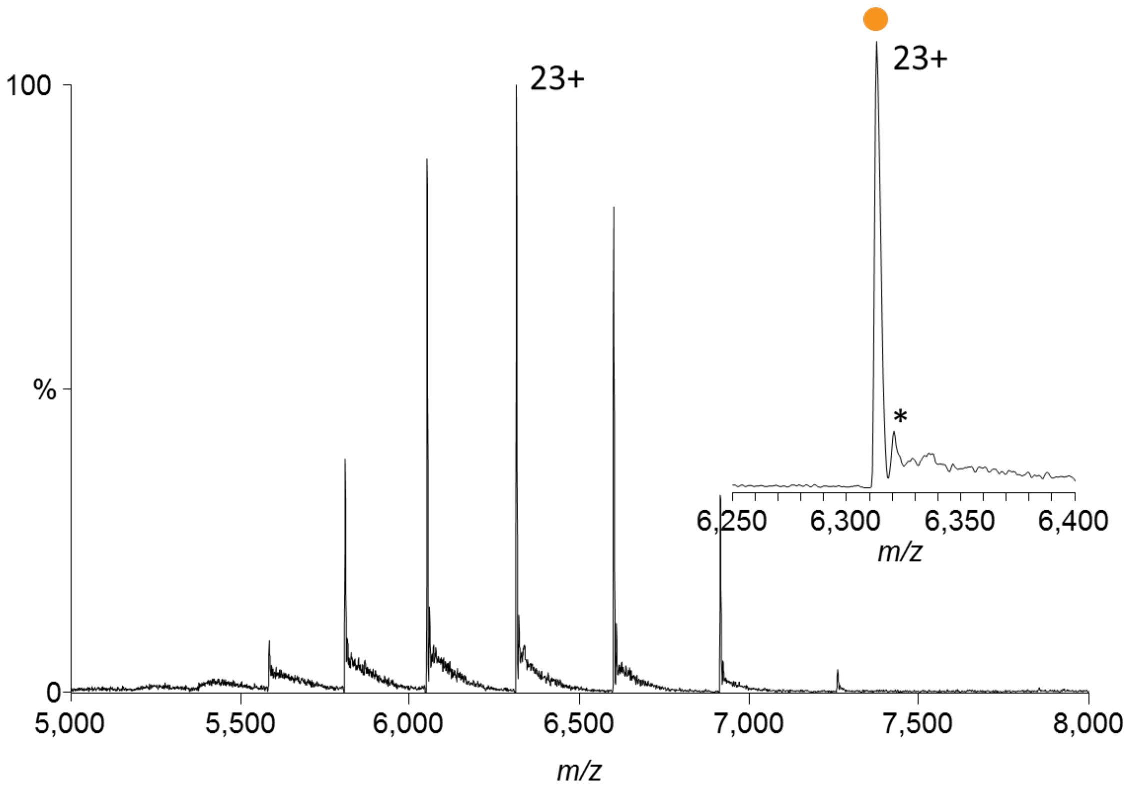

	dg-Her 2 ●	*
**M^{24+}**	6050.12	6057.10
**M^{23+}**	6313.15	6320.71
**M^{22+}**	6600.02	6608.06
**M.W. observed (Da)**	145178	145351
**M.W. S.D.**	1	5
**M.W. [dg-Her] theoretical (Da)**	145167	145343
**Abundance (%)**	87.7	12.3
**S.D. Abundance (%)**	1.5	1.5

Figure S3. MS/MS analysis of the azido-dg-Her 4 sample obtained using method A. The azide insertion was only observed on Q298$_{H}$, the glutamine closest to N300. The coverage obtained was 98% for the light chain and 86% for heavy chain.

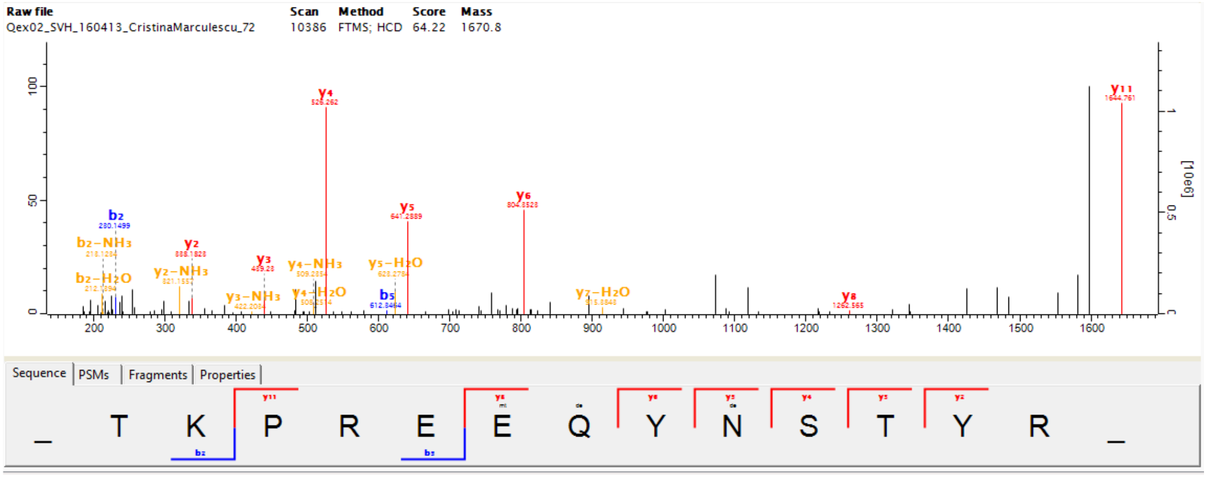

Figure S4. MS/MS analysis of the azido-dg-Her 4 sample obtained using method C. This indicates that the azide insertion occured at Q3$_{H}$.

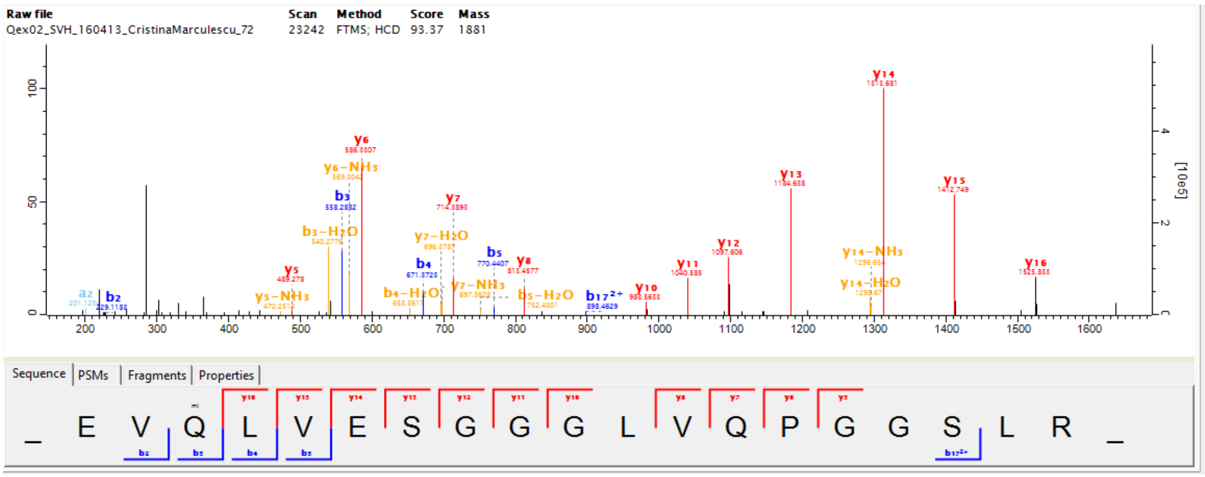

Figure S5. **nMS analysis of [DFO]$_2$-dg-Her 7**. (a) nMS analysis performed immediately after synthesis. The conjugation of DFO caused non-specific metal uptake and peak broadening. In this case, reasonable desolvation conditions reduced the majority of non-specific adducts leaving only minor peak tailing and allowing semi-quantitative analysis of conjugation products. Five major peaks were found and structures could be assigned to four of these as shown based on relative peak heights of Herceptin-derived products; (b) nMS analysis was performed 7 months after synthesis. In this case, the standard deviation is higher than in the previous spectrum due to broadening of the peaks; analysis suggested little or no degradation within the limits of estimation. We speculate that the species labelled * may be an impurity in the DFO chelation reagent but is in too low abundance to be more conclusive.

(a)

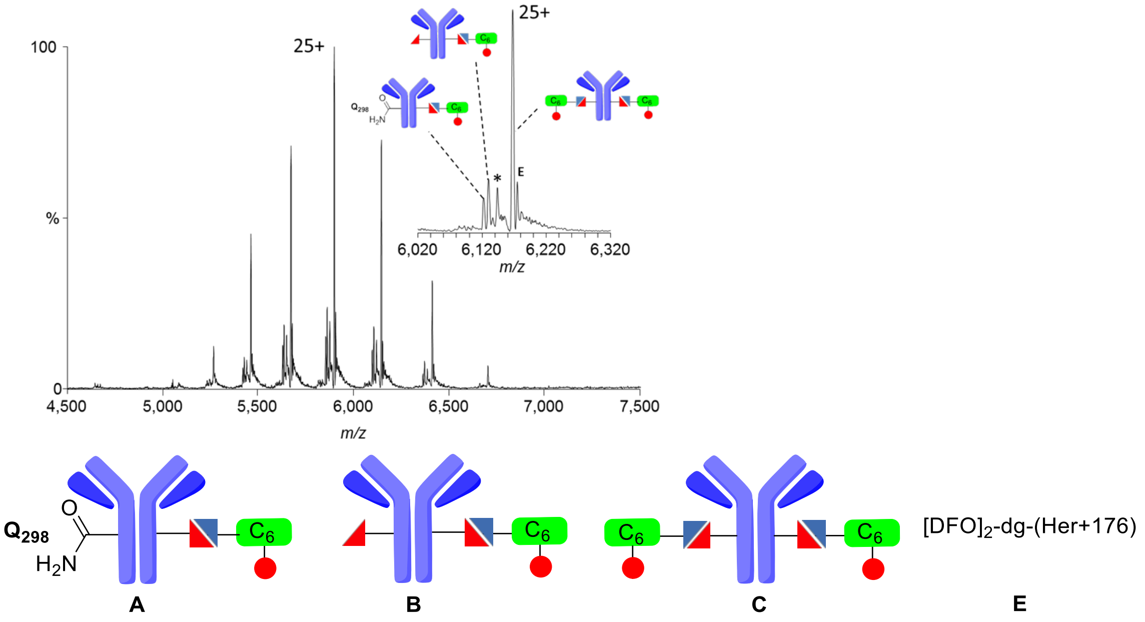

	A	B	*	C	E
**M/26**	5627.56	5634.76	5648.01	5671.24	5677.63
**M/25**	5852.3	5859.99	5873.8	5897.63	5904.83
**M/24**	6096.09	6104.65	6118.53	6143.8	6151.09
**M.W. observed (Da)**	146285	146480	146821	147423	147597
**M.W. S.D.**	5	7	1	6	5
**M.W. theoretical (Da)**	146247	146448	Unassigned	147323	147599
**Average abundance (%)**	10.4	15.5	12.7	61.4	Only Her species were considered, this is a conjugate

**S.D. Abundance**	0.5	0.5	0.7	1.5	of Her+176 antibody species.

(b)

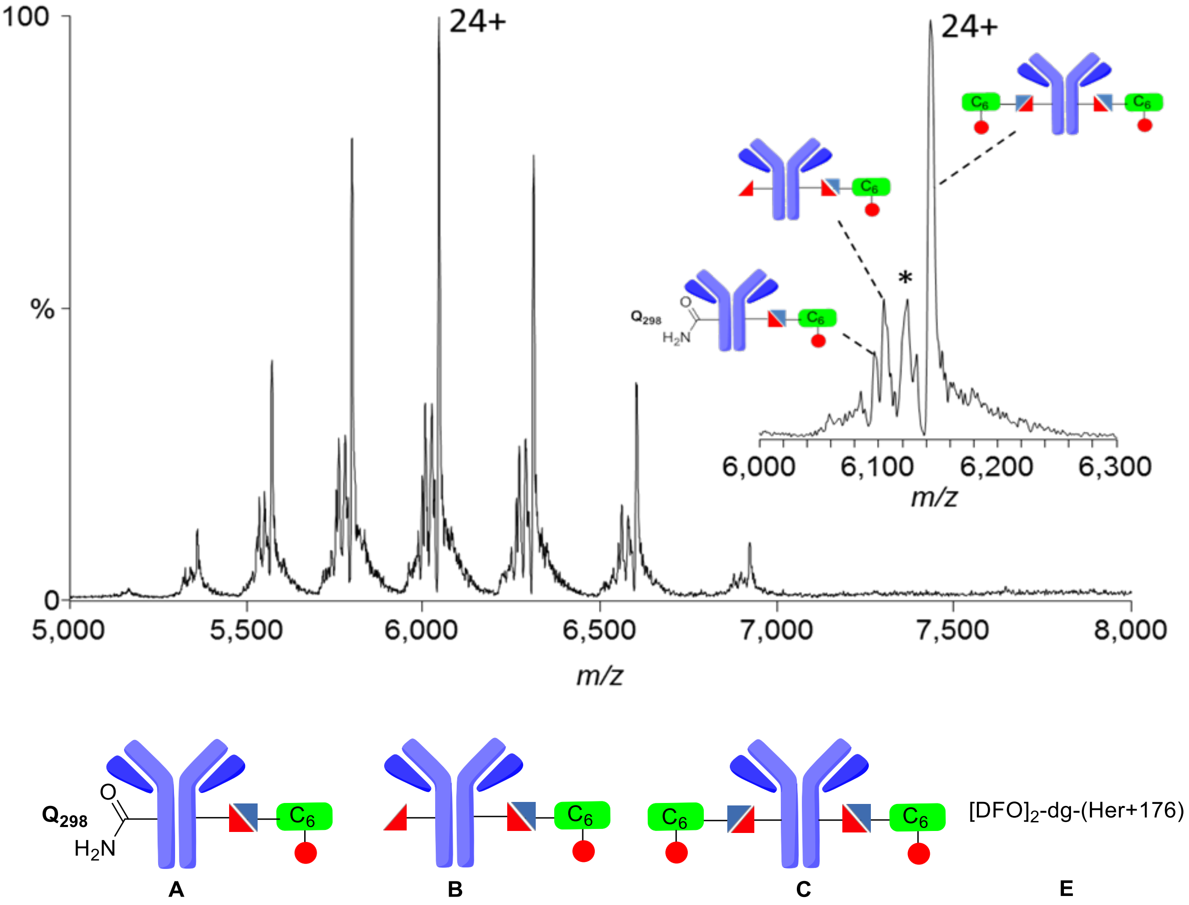

	A	B	*	C
**M/25**	5853.38	5860.05	5878.95	5897.95
**M/24**	6096.39	6104.29	6124.11	6143.41
**M/23**	6362.88	6370.59	6388.51	6411
**M.W. observed (Da)**	146307	146485	146939	147424
**M.W. S.D.**	17	13	23	6
**M.W. theoretical (Da)**	146247	146448	Unassigned	147323
**Average abundance (%)**	11.5	18.2	18.2	52.0
**Abundance S.D.**	0.4	0.2	0.2	0.8

Figure S6. Analysis of [DFO·IL1α]$_2$-dg-Her 8. (a) nMS analysis was performed immediately after synthesis. The conjugation of DFO caused non-specific metal uptake and peak broadening. In this case the nMS data is less well resolved and quantitative analysis is not possible because low abundance species cannot be resolved from poorly desolvated major products. The spectrum exhibits three major peaks, with the base peak **B** corresponding to the expected product, [DFO·IL1α]$_2$-dg-Her, suggesting **8** is the major product of conjugation. In addition, **A** can be assigned to the singly incorporated IL1α+DFO-alkyne and we postulate that ***** is a degradation product; (b) Postulated structure for *****, which could be a degradation product of **8**, formed *via* a retro-Michael elimination of the thiosuccinimide group (observed mass difference between ***** and **A** ≈ 427 Da); (c) nMS analysis performed 7 months after synthesis showing more extensive degradation.

(a)

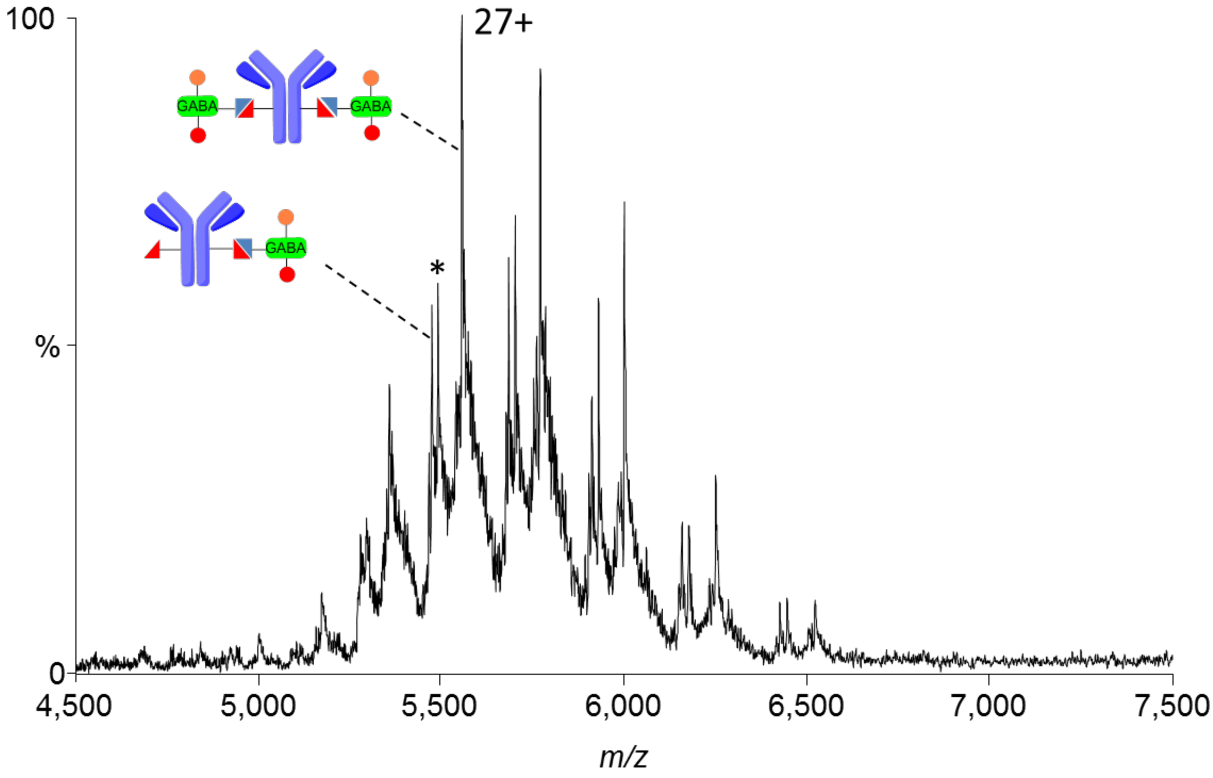

	A	*	B
**M/27**	5473.96	5490.63	5556.15
**M/26**	5684.02	5702.12	5770.79
**M/25**	5911.63	5930	6000.4
**M.W. observed (Da)**	147765	148225	149996
**M.W. S.D.**	6	5	16
**M.W. theoretical (Da)**	147758	See below	149943

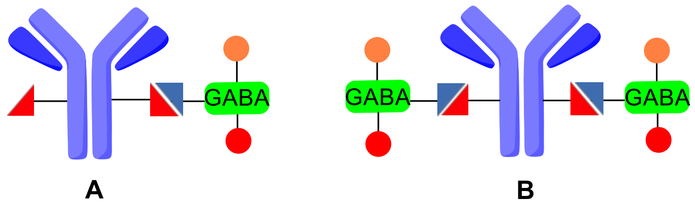

(b)

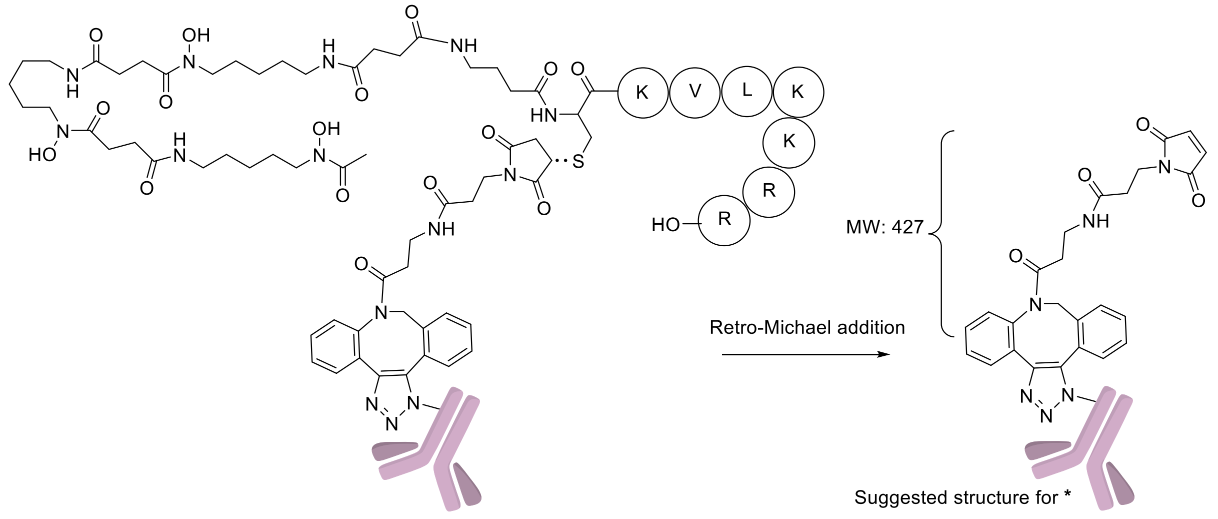

(c)

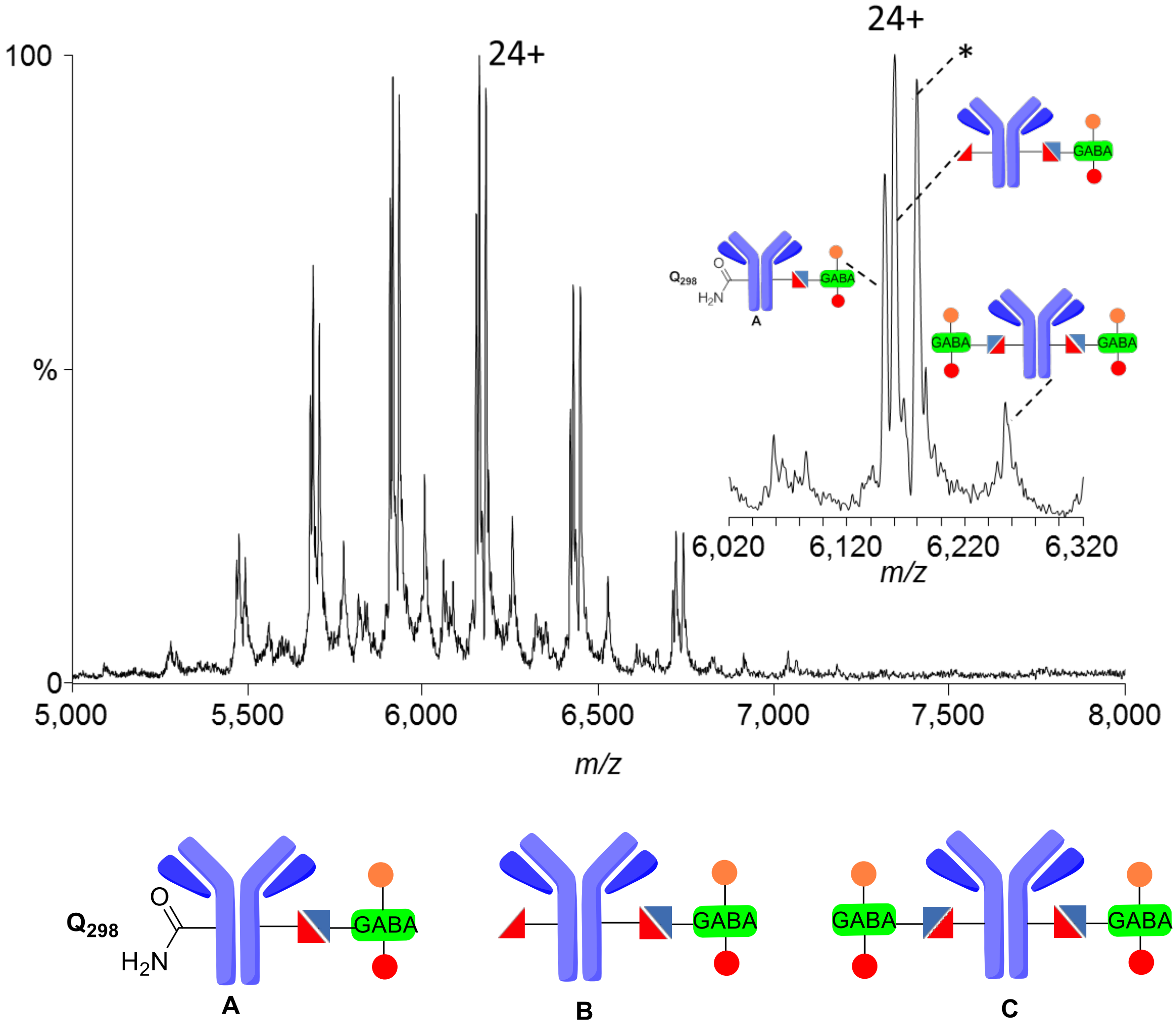

	A	B	*	C
**M/26**	5679.21	5686.52	5704.43	5773
**M/25**	5905.98	5913.78	5931.47	6003
**M/24**	6151.9	6160.29	6178.99	6253
**M.W. observed (Da)**	147626	147822	148274	150057
**M.W. S.D.**	6	2	14	13
**M.W. theoretical (Da)**	147557	147758	Unassigned	149943
**Average abundance (%)**	25.4	34.1	29.8	10.7
**S.D. Abundance**	1.4	0.5	2.2	1.4

Figure S7. Synthesis of randomly modified [DFO]$_{mix}$-Her 11[3]. (a) Schematic for synthesis of **11**; (b) Analysis of deconvoluted rLCMS for **11**.

(a)

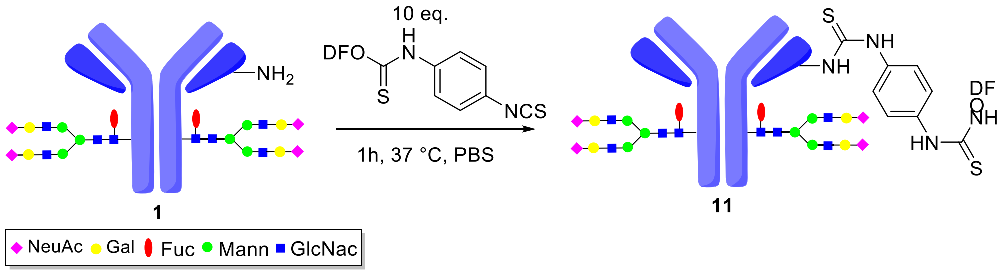

(b)

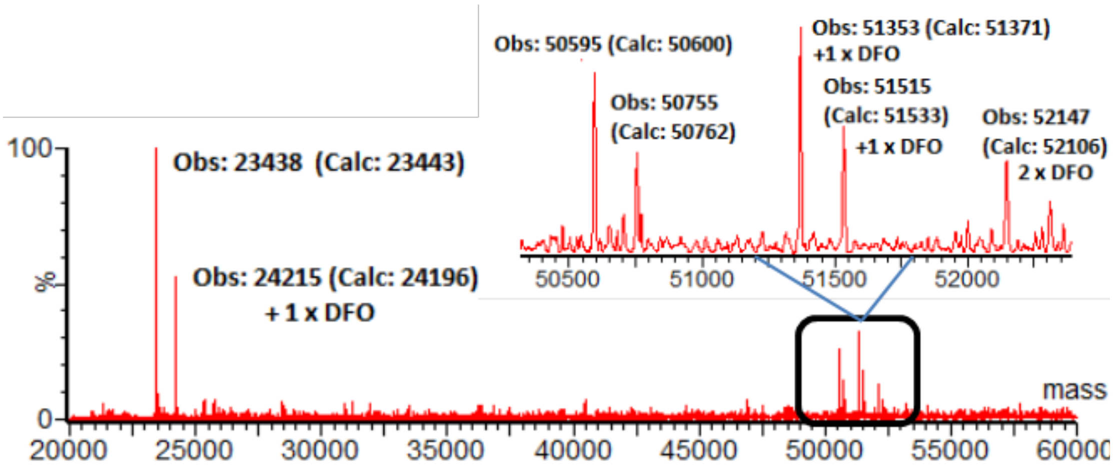

Figure S8. Saturation binding ELISA of wt-Her 1 and modified variants 7, 8 and 11. HER2-expressing MDA-MB-231/H2N cells were used as bait. K_D values for each conjugate were determined.

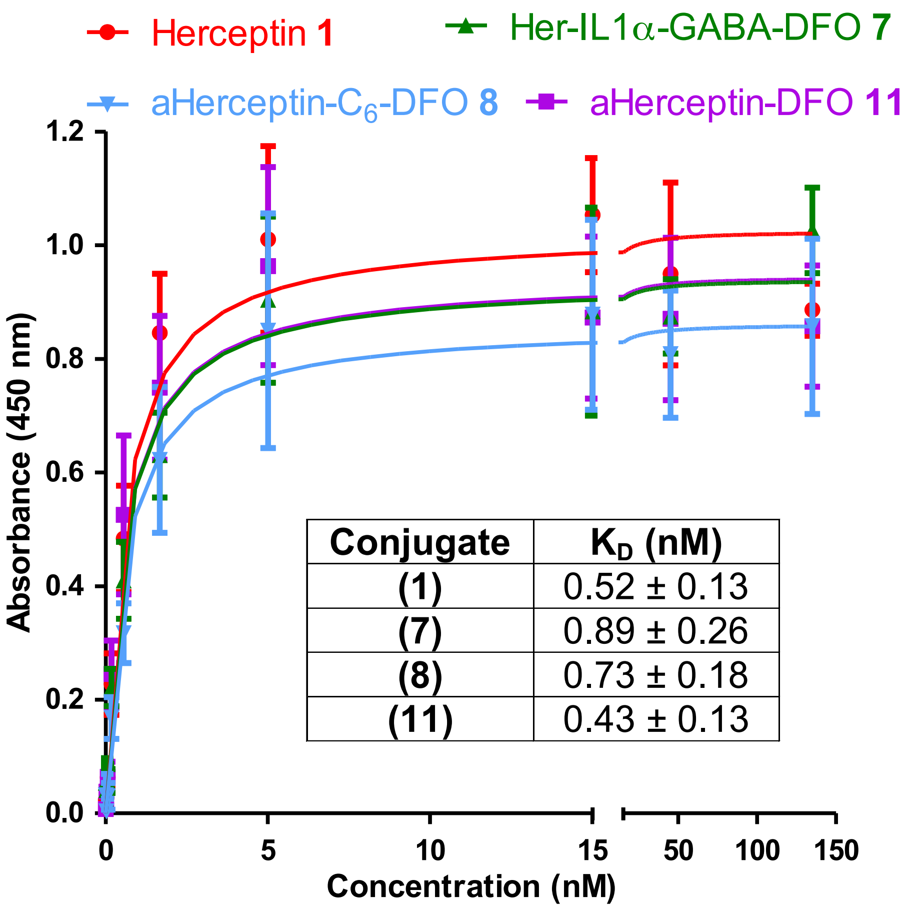

Figure S9. Stability of [^{89}Zr·DFO+IL1α]$_2$-dg-Her 10 in human serum as determined by iTLC

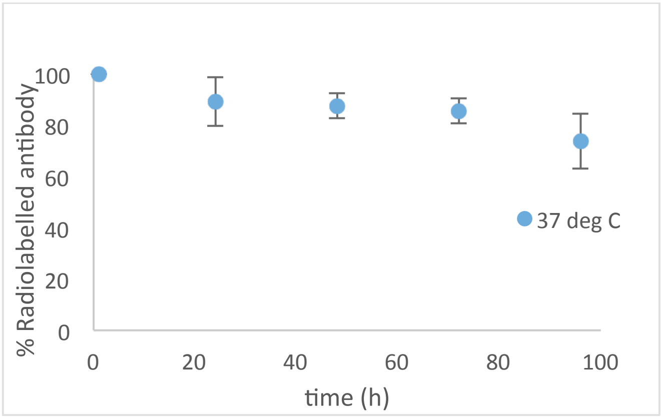

Figure S10. nMS analysis of TGase mediated azide incorporation into anti-γH2AX monoclonal antibody (05-636, clone JBW301, EMD Millipore). (a) Schematic for the azide incorporation into deglycosylated anti-γH2AX mouse monoclonal antibody; (b)Table showing reaction conditions and product distribution; (c) Graphical representation of product outcome; (d) nMS spectra of anti-γH2AX antibody conjugates. Based on these preliminary experiments, limits of regio-selectivity displayed by the 'Q-tag'-TGase method appear to be similar or even worse: compared with Herceptin, conversions of deglycosylated anti-γH2AX to azido-dg-anti-γH2AX are poorer, whilst still obtaining the triple addition product. We were not able to perform MS/MS experiments to map out the site of triple modification in the case of the anti-γH2AX antibody as this was commercially sourced (Merck Millipore, clone JBW301) and we did not have access to its sequence.

(a)

	Dg Ab concentration (mg/mL)	TGase (U)/mg Ab	N_3-PEG_3-NH_2 3 (eq.)	Time (h)
Reaction A	1	6	80	18
	S.M. (39%); monoaddition (41%); diaddition (16%); triaddition (4%)			
Reaction B	1	9 + 9 (after 17 h)	120	41
	S.M. (29%); monoaddition (44%); diaddition (23%); triaddition (4%)			
Reaction C	1	6 + 6 (after 17 h)	160	43
	S.M. (46%); monoaddition (40%); diaddition (12%); triaddition (2%)			
Reaction D	2.6	9 + 9 (after 16 h)	120	36
	S.M. (19%); monoaddition (40%); diaddition (30%); triaddition (11%)			

(b)

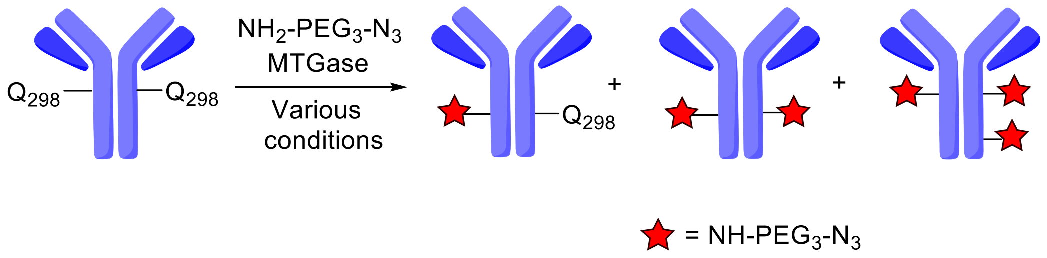

(c)

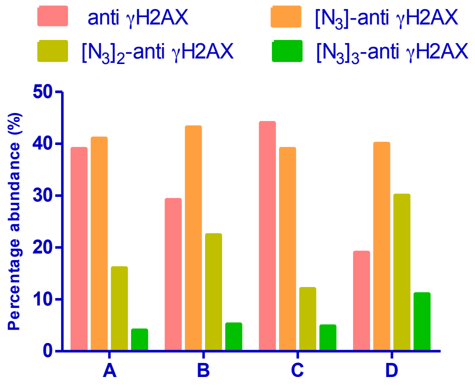

4. Materials and General Methods

Purification of the antibodies species was performed using 50 or 30 kDa MW cut-off Amicon Ultra-0.5 mL Centrifugal Filters (Millipore). PNGase F was purchased from New England Biolabs (NEB). 11-Azido-3,6,9-trioxaundecan-1-amine (Azido-PEG$_3$-amine) was purchased from Conju-Probe. DBCO-C$_6$-Acid **SI2** was purchased from Click Chemistry Tools. The Fmoc-protected IL1α peptide **SI3** was purchased from Cambridge Peptides. Deferoxamine mesylate salt, Cy3-DBCO **SI1** and benzocyclooctyne-maleimide (DBCO-Maleimide) were purchased from Sigma Aldrich. TGase, recombinant microbial (bacterial) transglutaminase (recombinantly produced in *E. coli*, gene derived from *Streptomyces mobaraensis*) was obtained from Zedira (code T001). Protease inhibitor tablets were purchased from Roche. The film and Phosphor Imager System (Cyclone plus) were both from Perkin Elmer. Perfect Protein Marker™ (EMD Millipore) was used as ladder for electrophoresis. Solvents for NMR experiments were purchased from Sigma Aldrich.

For NMR experiments, samples were dissolved in DMSO-d6 and analysed on Bruker 400 or 500 MHz spectrometers at room temperature. The chemical shifts, δ, are reported in ppm (parts per million). The residual solvent peaks have been used as an internal reference. The abbreviations for the peak multiplicities are as follows: s (singlet), d (doublet), dd (doublet of doublets), t (triplet), q (quartet), m (multiplet) and br (broad). Other abbreviations used were 'app', standing for apparent and 'ar', standing for aromatic.

5. Synthesis of antibody labelling reagents
5.1 Synthesis of DFO-alkyne 5

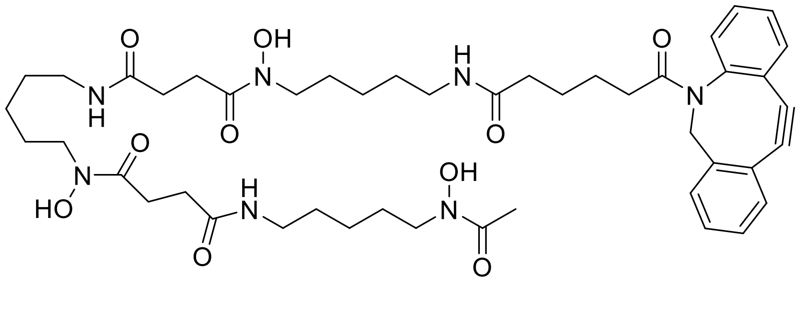

To a stirred solution of DBCO-C$_6$-acid **SI2** (13.5 mg, 0.04 mmol) in DMF (0.32 mL), were added HATU (23.0 mg, 0.06 mmol) and DIPEA (13.3 µL, 9.80 mg, 0.08 mmol). Separately, deferoxamine (DFO) mesylate salt (26.3 mg, 0.04 mmol) was dissolved in DMF (0.32 mL) and DIPEA (13.3 µL, 9.80 mg, 0.08 mmol) and NMM (30.0 µL) were added. Both solutions were stirred at room temperature for 1 h. During this time, the solution containing DBCO-C$_6$-acid **SI2** turned orange. After 1 h, the DFO-mesylate salt solution was added to the DBCO-C$_6$-acid solution and the reaction mixture stirred for 2 days, at room temperature, under Nitrogen. After this time, ice cold acetone (5 mL) was added and the reaction mixture was sonicated for 5

min. This resulted in a white solid which was separated from the liquid by centrifugation (7000 rpm). The white solid was then washed with acetone (2 x 5 mL) and water (3 x 5 mL), with sonication and centrifugation in between the washes. Finally the resulting white solid was dried under high vacuum to afford 12 mg (0.03 mmol, 69%) of **5**. ^{1}H NMR (400 MHz, DMSO-d6) 9.65 (1H, br s, OH), 9.60 (2H, br s, 2 x OH), 7.78 (2H, app. t, J= 6.4 Hz, 2 x NH), 7.63-7.56 (3H, m, 2 x H_{ar} and NH), 7.51-7.44 (3H, m, 3 x H_{ar}), 7.40-7.32 (4H, m, 2 x H_{ar}), 7.29 (1H, dd, *J* = 7.3, *J* = 1.5 Hz, H_{ar}), 5.03 (1H, d, *J* = 14.3 Hz, C*H*H-DBCO), 3.60 (1H, d, *J* = 14.0 Hz, CH*H*-DBCO), 3.46-3.41 (6H, m, 3 x CH_2), 3.02-2.97 (4H, m, 2 x CH_2), 2.94-2.89 (2H, m, CH_2), 2.59-2.55 (4H, m, 2 x CH_2), 2.28-2.24 (4H, m, 2 x CH_2), 2.16 (1H, m, C*H*H), 1.96 (3H, s, CH_3), 1.84-1.80 (2H, m, CH_2), 1.75 (1H, m, CH*H*), 1.53-1.44 (6H, m, 3 x CH_2), 1.41-1.28 (8H, m, 4 x CH_2), 1.24-1.15 (8H, m, 4 x CH_2); ^{13}C NMR (125 MHz, DMSO-d6) 132.9, 129.8, 129.4, 128.6, 128.5, 128.1, 127.3, 125.6, 122.9, 121.9, 114. 8, 108.7, 72.7, 55.2, 47.5, 47.2, 38.9, 38.7, 35.5, 34.4, 30.3, 29.3, 28.0, 26.5, 25.2, 24.9, 23.9, 20.9; IR: 3303 (w), 2930 (w), 2857 (w), 1714 (m), 1618 (s), 1563 (m), 1268 (s), 1197 (s), 722 (s); MS (ESI^{+}): *m/z*: 876.4 ([M+H]$^{+}$); HRMS (ESI^{+}) calculated for [$C_{46}H_{65}O_{10}N_7$ + H^{+}]: 876.48657, measured 876.48663.

^{1}H-NMR (DMSO-d6)

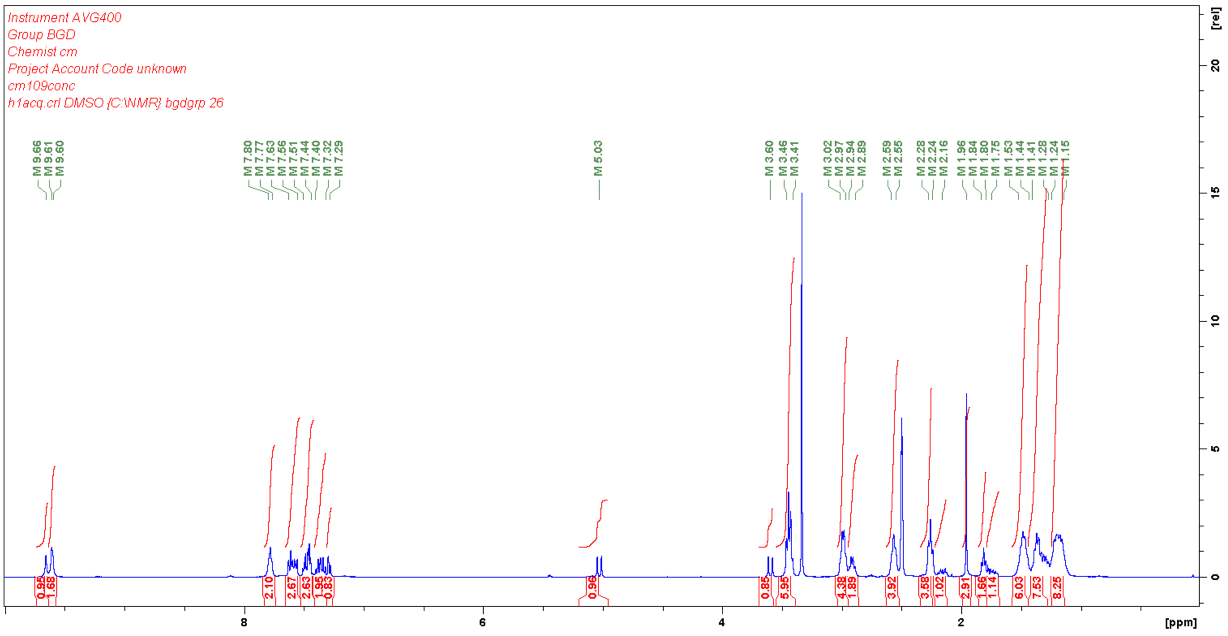

^{13}C-NMR (DMSO-d6)

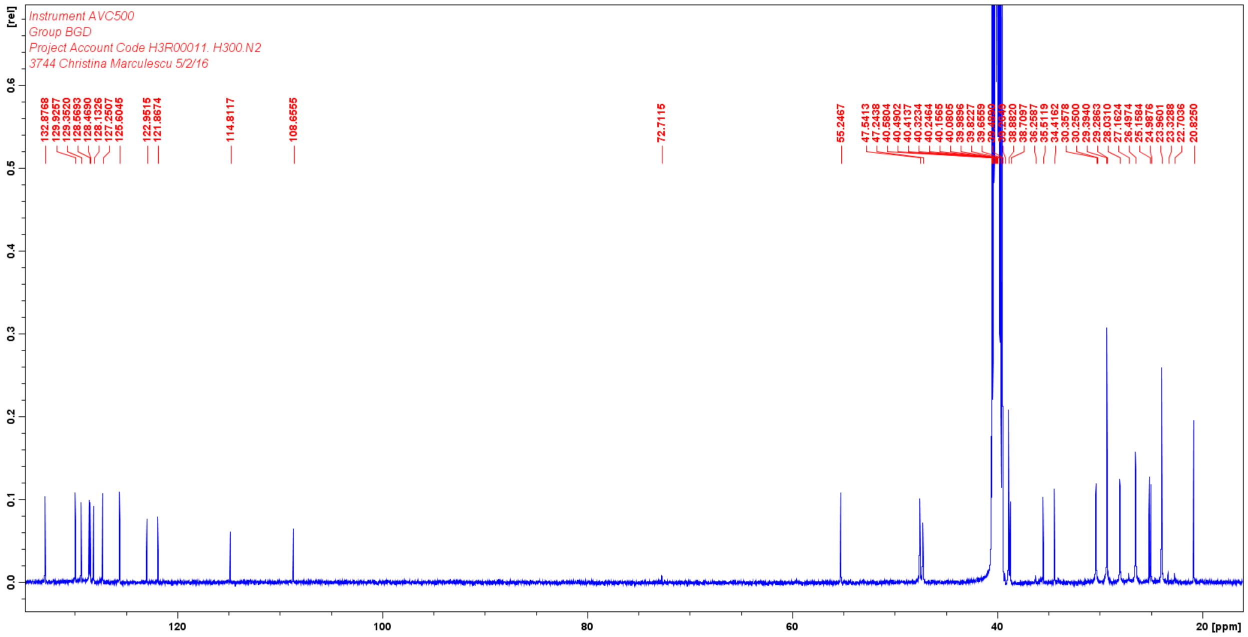

Analytical HPLC-MS analysis was achieved using an eluent A: 0.1% formic acid acid; eluent B: acetonitrile, using a flow rate of 0.5 mL/min and a gradient of 10-90 % B in 7 min, returning to start conditions over 0.1 min with an 11 min run time. A flow splitter constructed of a short length of 0.0007 inch i.d. PEEK tubing attached to a T-fitting on the mass spectrometer inlet diverted ~ half of this to waste, the remainder going to the mass spectrometer detector. Flow was diverted away from the mass spectrometer at the beginning and end of each chromatogram. Mass spectrometry settings were; capillary voltage was set at 3.2 kV, extractor lens 2V, RF lens 0.3V, desolvation gas flow 450 L/h, cone gas flow 90 L/h, source temperature 120 °C, and desolvation temperature 425 °C. DFO-alkyne **5** was monitored using total ion count in electrospray positive more from m/z 250-1000 with a cone voltage of 10V (retention time 7.18 min) or UV absorbance at 215 nm (elution time 7.14 min).

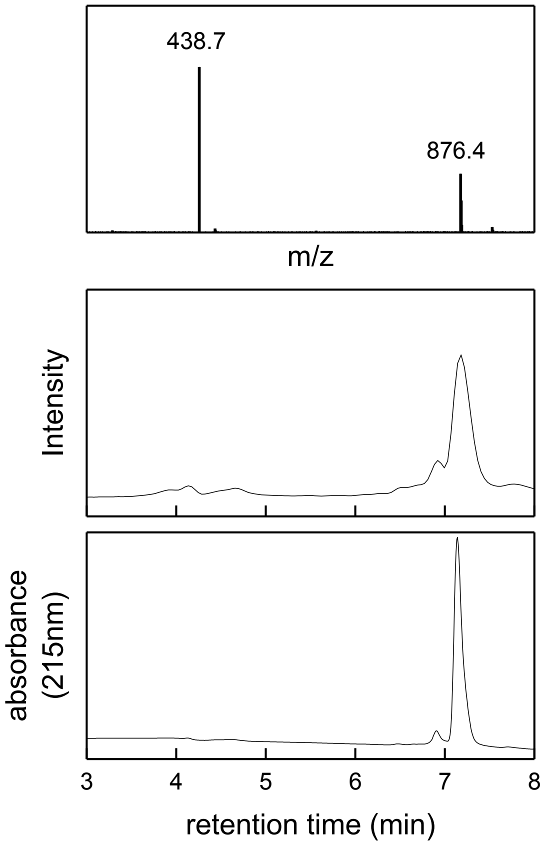

5.2 Synthesis of *N*-succinyldeferoxamine, DFO-C_3-COOH SI4

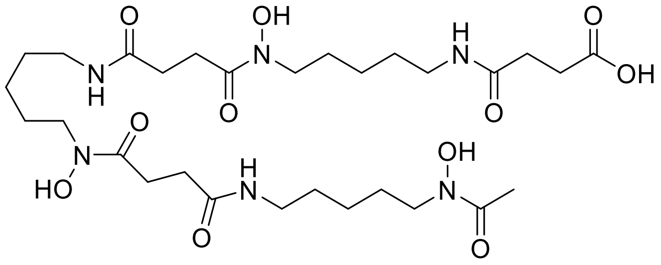

To a stirred mixture of deferoxamine (DFO) mesylate salt (200 mg, 0.30 mmol) and succinic anhydride (40.0 mg, 0.40 mmol) in 20 mL DMF, was added triethylamine (96 µL, 0.70 mmol) under Argon. After 42 h of stirring at room temperature, the solvent was removed *in vacuo*. The resulting solid was washed with acetone (3 x 10 mL) and diethyl ether (3 x 10 mL) to afford 193 mg of (0.29 mmol, 97%) of **SI4** as a white solid. Analytical HPLC-MS analysis was achieved using an eluent A: 0.1% trifluoroacetic acid; eluent B: acetonitrile, using a flow rate of 0.5 mL/ min and a gradient of 10 – 90 % B over 5 min, returning to starting conditions in 0.1 min with a run time of 10 min. A flow splitter constructed of a short length of 0.0007 inch i.d. PEEK tubing attached to a T-fitting on the mass spectrometer inlet diverted ~ half of this to waste, the remainder going to the mass spectrometer detector. Flow was diverted away from the mass spectrometer at the beginning and end of each chromatogram. Mass spectrometry settings were; capillary voltage was

set at 3.2 kV, extractor lens 2V, RF lens 0.3V, desolvation gas flow 450 L/h, cone gas flow 90 L/h, source temperature 120 °C, and desolvation temperature 425 °C. DFO-C_3-COOH **SI4** was monitored using total ion count in electrospray positive more from m/z 250-1000 with a cone voltage of 10V (retention time 4.01 min) and UV absorbance at 215 nm (elution time 4.07 min).

^{1}H NMR (500 MHz, DMSO-d6) 12.05 (1H, br s, COO*H*), 9.65 (1H, br s, OH), 9.60 (2H, br s, 2 x OH), 7.81-7.76 (3H, m, 3 x NH), 3.44 (6H, t, *J* = 7.3 Hz, 3 x CH_2), 2.99 (6H, q, *J* = 6.6 Hz, 3 x CH_2), 2.57 (4H, t, J = 7.3 Hz, 2 x CH_2), 2.40 (2H, t, *J* = 7.3 Hz, CH_2), 2.30-2.25 (6H, m, 3 x CH_2), 1.96 (3H, s, CH_3), 1.52-1.46 (6H, m, 3 x CH_2), 1.40-1.34 (6H, m, 3 x CH_2), 1.24-1.18 (6H, m, 3 x CH_2); ^{13}C NMR (125 MHz, DMSO-d6) 174.3, 172.4, 171.8, 171.2, 170.6, 47.5, 47.2, 38.9, 30.5, 30.4, 29.7, 29.3, 28.0, 26.5, 23.9, 20.8; IR: 3300 (w), 3098 (w), 1618 (s), 1563 (m), 1549 (m), 1196 9m); MS (ESI$^+$) *m/z*: 661.5 ($[M+H]^+$); HRMS (ESI$^+$) calculated for $[C_{29}H_{52}O_{11}N_6 + H^+]$: 661.37668, measured 661.37616; m.p.: 150 -152 °C .

^{13}C-NMR (DMSO-d6)

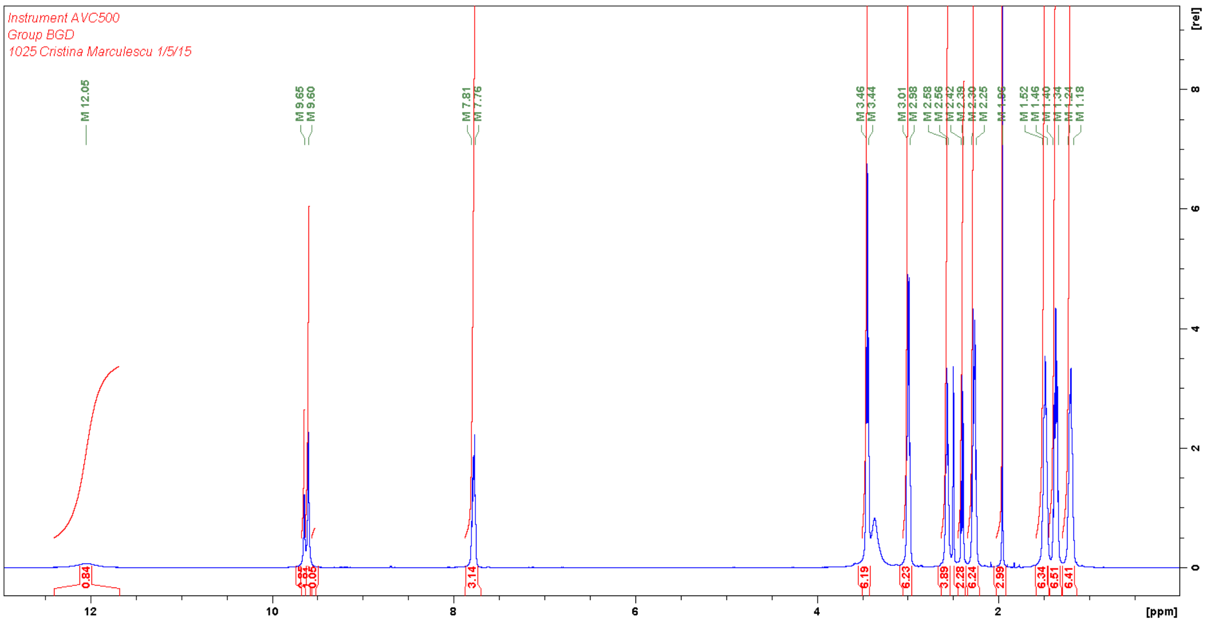

^{13}C-NMR (DMSO-d6)

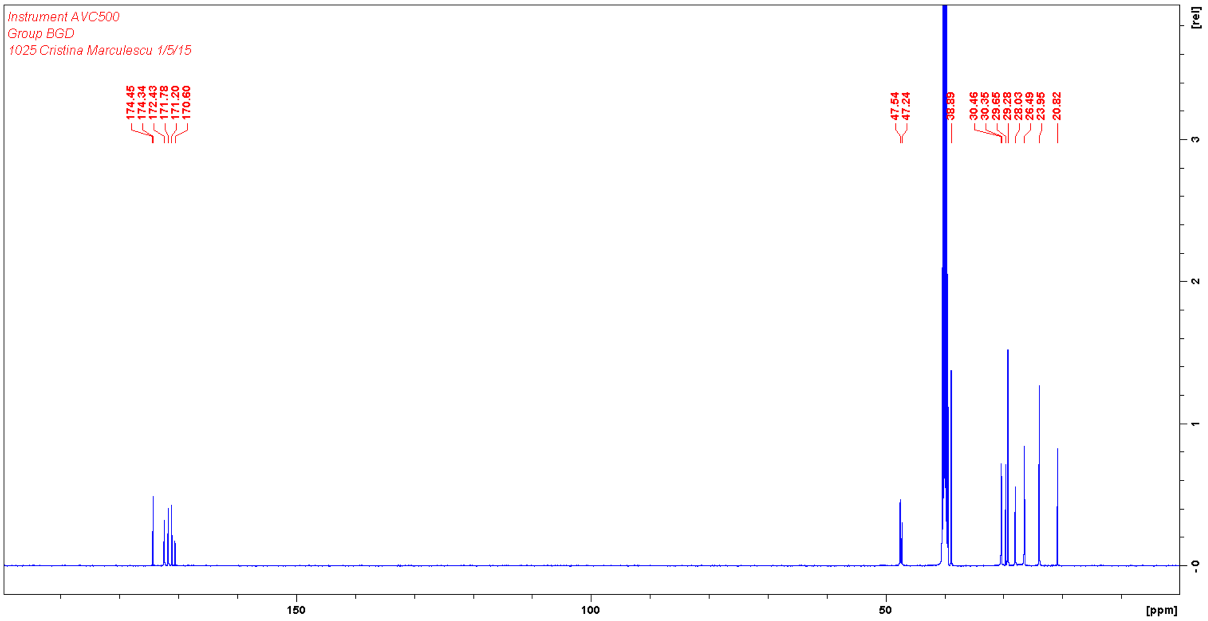
Instrument AVC500
Group BGD
1025 Cristina Marculescu 1/5/15
174.45
174.34
172.43
171.78
171.20
170.60
47.54
47.24
38.89
30.46
30.35
29.65
29.28
28.03
26.49
23.95
20.82
150
100
50
[ppm]
[rel]

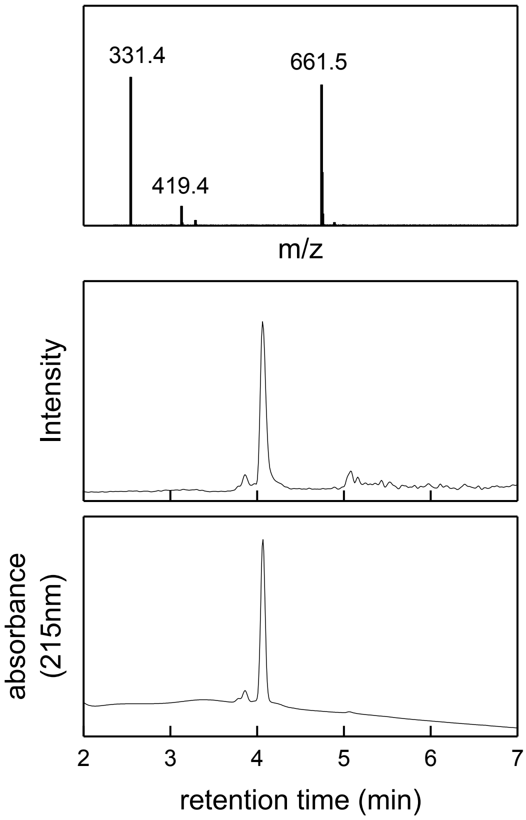
331.4
419.4
661.5
m/z
Intensity
absorbance
(215nm)
2
3
4
5
6
7
retention time (min)

5.3 Synthesis of IL1α-GABA-DFO SI5

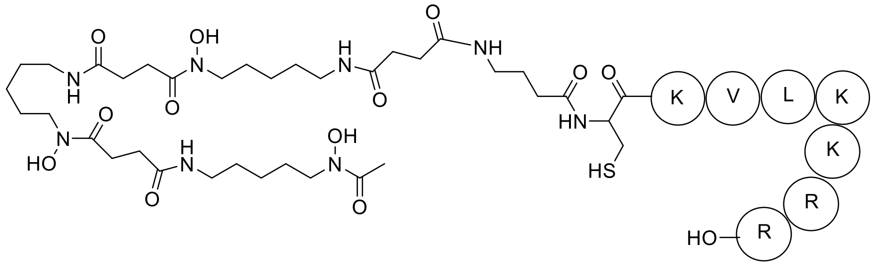

FMOC-C(Trt)K(Boc)VLK(Boc)K(Boc)R(Pbf)R(Pbf)-Wang resin **SI3** (150 mg, 0.13 mmol/g) was swollen with DMF (5 mL), Fmoc deprotected with 20% piperidine in DMF (8 mL) for 45 min at room temperature and then washed with DMF (2 x 10 mL). The resin was then incubated (with shaking) with Fmoc-gamma aminobutyric acid (Fmoc-GABA) (31.7 mg, 97.5 μmol, 5 eq.), HATU (22.9 mg, 97.5 μmol, 5 eq.) and DIPEA (17 μL, 97.5 μmol, 5 eq.) in DMF (3 mL) for 1.5 h, at room temperature. After this time the resin was washed with DMF (10 mL), DCM (10 mL) and DMF (10 mL) and a second portion of the coupling cocktail (Fmoc-GABA, HATU, DIPEA- same amounts as before) was added for another 1.5 h. After washing the resin with DMF (20 mL) and DCM (20 mL), Fmoc deprotection of the *N*-terminus was performed by incubating the resin with 20% piperidine in DMF (5 mL) for 45 min at room temperature. Next, *N*-succinyldeferroxamine **SI4** was coupled by incubating the resin (with shaking) with a mixture of **SI4** (64 mg, 97.5 μmol, 5 eq.), HATU (22.9 mg, 97.5 μmol, 5 eq.) and DIPEA (17 μL, 97.5 μmol, 5 eq.) in DMF (3 mL) for 1.5 h, at room temperature. After this time the resin was washed with DMF (10 mL), DCM (10 mL) and DMF (10 mL) and a second portion of the coupling cocktail (**SI4**, HATU, DIPEA) was added for another 1.5 h. After washing the resin with DMF (20 mL) and DCM (20 mL), the resin was dried *in vacuo*. Full deprotection and cleavage off the resin was achieved by treating the resin with a mixture of TFA : EDT : TIS = 95 : 2.5 : 2.5 (v/v/v) for 2 h, at room temperature. HPLC purification was performed using an RPB C18 (5 μm, 3.2 x 100 mm, Hichrom, UK) column maintained at 30 °C with eluent A: 10 mM formic acid and eluent B: acetonitrile, using a flow rate of 0.5 mL/ min and a gradient of 10-30% B over 7 min, 30-100% over 1 min, and returning to starting conditions in 0.1 min with a total run time of 12 min. The compound was collected using a rhenodyne valve to divert the flow from waste to the collection vessel. Acetonitrile was removed from the collected fractions under vacuum and then the remaining solvent was lyophilised overnight. For analysis of the pure sample the gradient was changed to 5-100 % B in 7 min, returning to start conditions over 0.1 min with an 11 min run time. A flow splitter constructed of a short length of 0.0007 inch i.d. PEEK tubing attached to a T-fitting on the mass spectrometer inlet diverted ~ half of this to waste, the remainder going to the mass spectrometer detector. Flow was diverted away from the mass spectrometer at the beginning and end of each chromatogram. Mass spectrometry settings were; capillary voltage was set at 3.2 kV, extractor lens 2V, RF lens 0.3V, desolvation gas flow 450 L/h, cone gas flow 90 L/h, source temperature 120

°C, and desolvation temperature 425 °C. IL1α-GABA-DFO **SI5** (MW= 1757.1 Da), ([M+2H$^+$]/2 = 879.5; [M+3H$^+$]/3 = 586.7; [M+ 4H$^+$]/4 = 440.3) was monitored using total ion count in electrospray positive more from m/z 250-1000 with a cone voltage of 10V (retention time 4.15 min) and UV absorbance at 215 nm (elution time 4.12 min). HRMS (ESI+) calculated for [$C_{77}H_{144}O_{20}N_{24}S$ + H$^+$]: 1758.07822, measured 1758.07898.

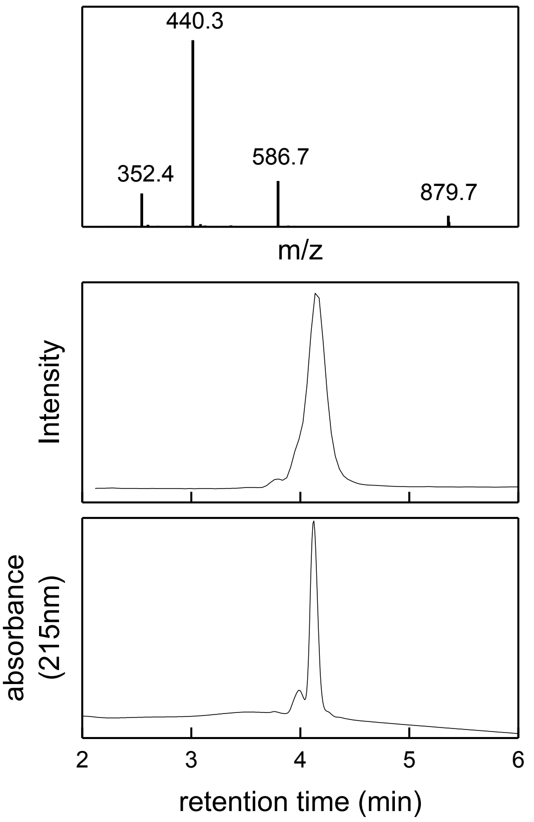

5.4 Synthesis of DFO+IL1α-alkyne 6

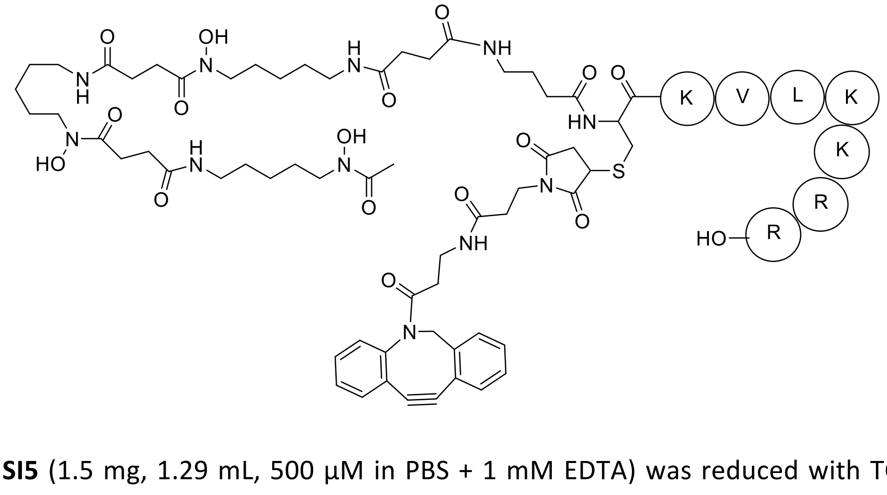

IL1α-GABA-DFO **SI5** (1.5 mg, 1.29 mL, 500 µM in PBS + 1 mM EDTA) was reduced with TCEP (15.5 µL, 1.2 eq., 50 mM stock in PBS + 1 mM EDTA) for 1 h at room temperature and then DBCO-maleimide (19.3 µL, 3 eq.,100 mM stock in DMSO) was added. The reaction was left to stand at room temperature for 1 h. After

this time, LC-MS analysis indicated that the reaction was complete. HPLC purification was achieved using an RPB C18 (5 µm, 3.2 x 100 mm, Hichrom, UK) column maintained at 30 °C with eluent A: 10 mM formic acid and eluent B: acetonitrile, using a flow rate of 0.5 mL/ min and a gradient of 10 – 80 % B over 7 min, returning to starting conditions in 0.1 min with a run time of 11 min. The peptide was collected using a rhenodyne valve to divert the flow from waste to the collection vessel. Acetonitrile was removed from the collected fractions *in vacuo* and the remaining solvent was lyophilized overnight. For analysis of the pure sample the gradient was 10-80 % B in 7 min, returning to start conditions over 0.1 min with an 11 min run time. A flow splitter constructed of a short length of 0.0007 inch i.d. PEEK tubing attached to a T-fitting on the mass spectrometer inlet diverted ~ half of this to waste, the remainder going to the mass spectrometer detector. Flow was diverted away from the mass spectrometer at the beginning and end of each chromatogram. Mass spectrometry settings were; capillary voltage was set at 3.2 kV, extractor lens 2V, RF lens 0.3V, desolvation gas flow 450 L/h, cone gas flow 90 L/h, source temperature 120 °C, and desolvation temperature 425 °C. IL1α+DFO-alkyne **6** (MW = 2185.67 Da) eluted as a Fe adduct ($[M+Fe+5H^+]/5$ = 448.6; $[M+Fe+4H^+]/4$ = 560.5, $[M+Fe+3H^+]/3$ = 747.1) adduct at 4.95 min (UV absorbance at 215 nm). HRMS (ESI^+) calculated for $[C_{102}H_{165}O_{24}N_{27}S + H^+]$: 2185.23143, measured 2185.24194.

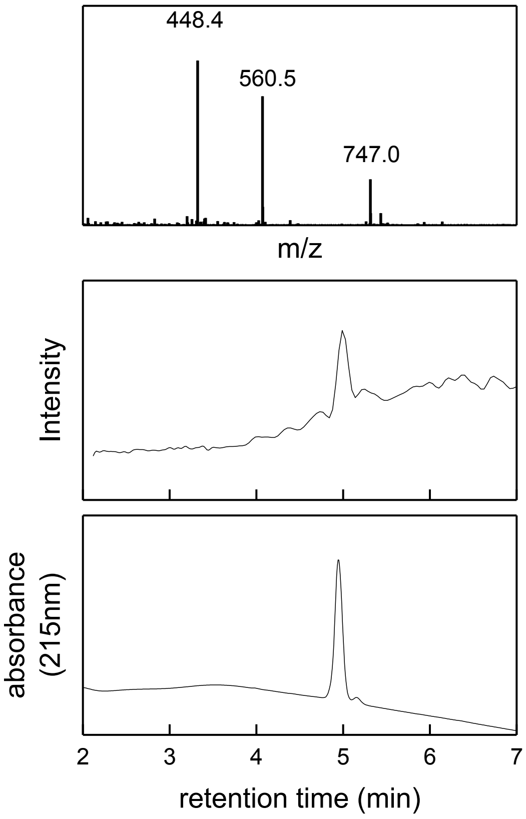

6. Chromatography Methods

Samples were analysed by HPLC using a separation module (Waters 2695, Watford, UK) equipped with a mass spectrometry detector (Waters micromass ZQ) and a Waters 2996 photodiode array detector. Compounds were monitored using total ion count in electrospray positive mode from *m/z* 250-1500 with a cone voltage of 10V or UV absorbance at 215 nm. Mass spectrometry settings were: capillary voltage was set at 3.2 kV, extractor lens 2V, RF lens 0.3V, desolvation gas flow 450 L/h, cone gas flow 90 L/h, source temperature 120 °C, and desolvation temperature 425 °C.

7. Antibody Conjugation Methods

7.1 LCMS analysis under reducing conditions

Aliquots (2 µg of antibody) from the reaction mixture were diluted with DTT (20 mM solution) to achieve a concentration of 0.02-0.1 mg/mL. The mixture was heated to 60 °C for 3 min and analysed on Chromolith RP-18e 5-2 mm HPLC guard cartridge (Merck) attached to a Waters 1525µ binary HPLC pump. Samples were eluted using buffers A (0.1% aqueous solution of formic acid) and B (acetonitrile). Prior to sample injection, the column was pre-treated as follows: 1 min 40% A, 0.6 mL/min followed by 20 seconds at 95% A, 0.4 mL/min followed by 40 seconds 95% A, 0.3 mL/min. Samples (5 µL) were injected onto the column via a Waters 2777C sample manager, and were eluted using a solvent gradient as follows: 30 seconds of 95% buffer A (flow rate 0.3 mL/min) followed by a 1 minute gradient to 40% buffer A (flow rate 0.3 mL/min) and hold at 40% A for a further 1.5 minutes. The flow rate was then increased to 0.75 mL/min for 30 seconds before a final wash using 2% A for 30 seconds. Eluted samples were injected into an LCT Premier XE mass spectrometer (Waters) operating in positive mode (ES+) with the analyser in 'V' mode. The capillary was set at 3000V and sample cone at 100V. Desolvation temperature was 150 °C. Collected data was analysed using MassLynx V4.1 software. Ion envelopes were deconvoluted using MaxEnt software. Minimum intensity ratios were set at 33% for both left and right. Raw data was not subjected to subtraction, smoothing or centring prior to deconvolution.

7.2 Synthesis and analysis

wt-Her 1

Light chain: Theoretical mass 23443 Da; Observed mass: 23441 Da
Heavy chain: Theoretical mass 50600 (G0F), 50762 (G0F) Da; Observed mass: 50601 (G0F), 50762 (G1F) Da

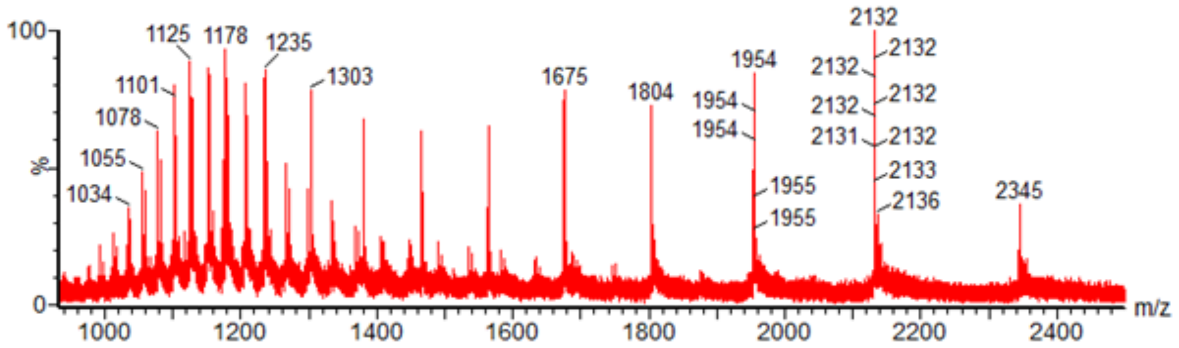

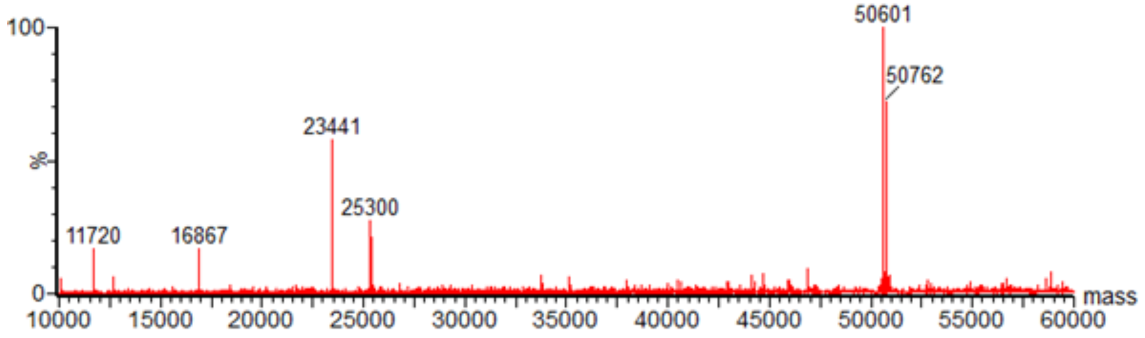

Herceptin deglycosylation

To a solution of wt-Her **1** (4.0 mg, 1.6 mL, 2.5 mg/mL in PBS) were added GlycoBuffer 2 (10X, part of PNGase Kit) (180 µL) and PNGase F (20 µL, 2500 U/mg Ab). The reaction mixture was left at 37 °C for 16 h, with shaking (250 rpm). After confirming (SDS-PAGE and LC-MS) that the reaction reached completion, the antibody was purified using 50 MW kDa centrifugal filters by repeated washes (4x) with PBS.

Light chain: Theoretical mass 23443 Da; Observed mass: 23441 Da

Heavy chain: Theoretical mass 49156 Da; Observed mass: 49156 Da

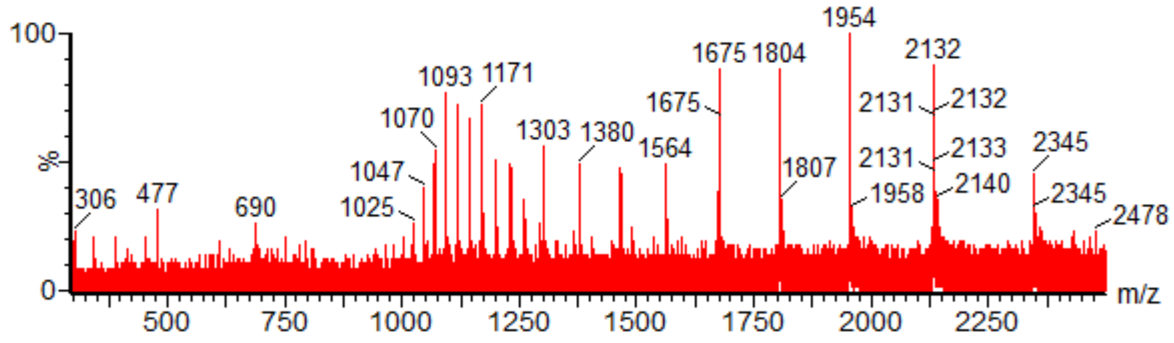

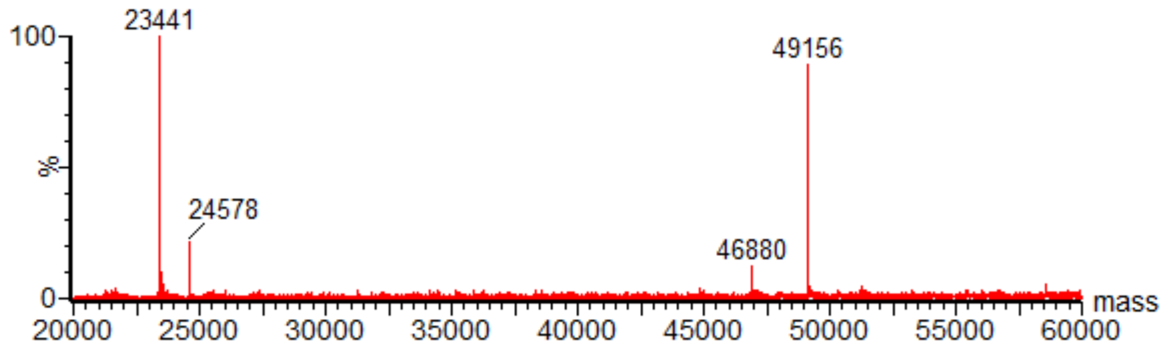

Azide incorporation into deglycosylated Herceptin

To a solution of deglycosylated Herceptin **2** in PBS were added polyetherazidoamine **3** and TGase enzyme (Supplementary Table S1). The reaction was left at 37 °C with shaking (250 rpm). In some reactions a second portion of polyetherazidoamine **3** and TGase enzyme and the reaction was further kept under the same conditions for another 2 h. After this time, LCMS analysis under reduced conditions indicated that the reaction reached completion. The azido-dg-Her **4** antibody conjugate was purified using 50 MW kDa centrifugal filters by repeated washes with PBS. Additionally, native mass spec analysis of the sample was performed to determine its purity.

Light chain: Theoretical mass 23443 Da; Observed mass: 23442 Da

Heavy chain: Theoretical mass 49357 Da; Observed mass: 49357 Da

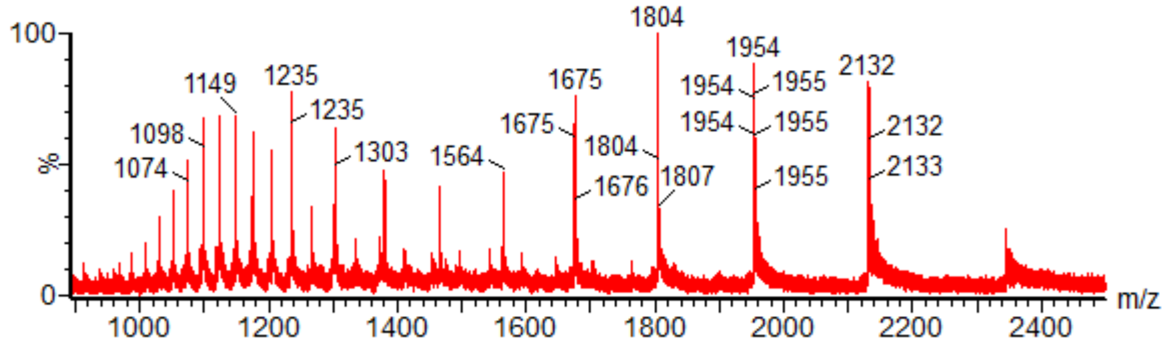

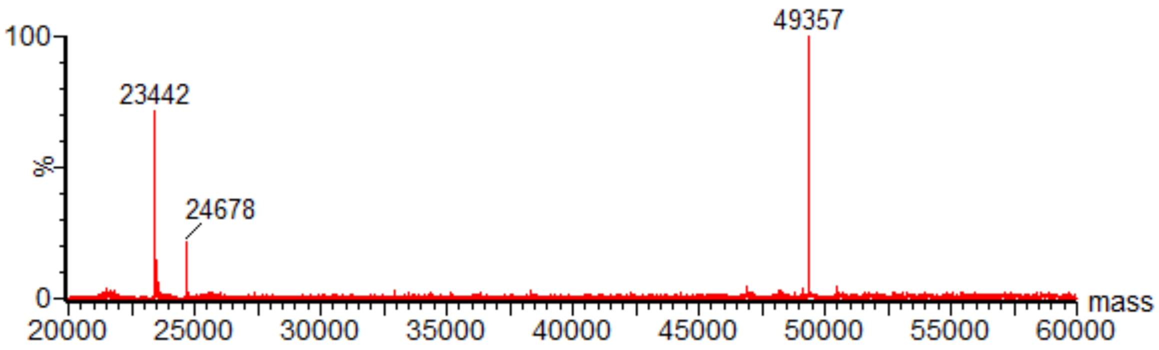

Synthesis of [Cy3]$_2$-dg-Her

To a solution of azido-dg-Her **4** (25 µL, 1.07 mg/mL in PBS) was added DBCO-Cy3 **SI1** (1.1 µL, 10 eq., 2 mg/mL in PBS) and the reaction mixture was left at 25 °C for 18 h, with shaking (250 rpm). After confirming by SDS-PAGE, and LCMS under reducing conditions that the reaction reached completion, [Cy3]$_2$-dg-Her antibody conjugate was purified using 30 MW kDa centrifugal filters by repeated washes with PBS.

Deconvoluted rLCMS data showing the light (LC) and heavy chain (HC) of [Cy3]$_2$-dg-Her

Light chain: Theoretical mass 23443 Da; Observed mass: 23443 Da

Heavy chain: Theoretical mass 50338 Da; Observed mass: 50340 Da

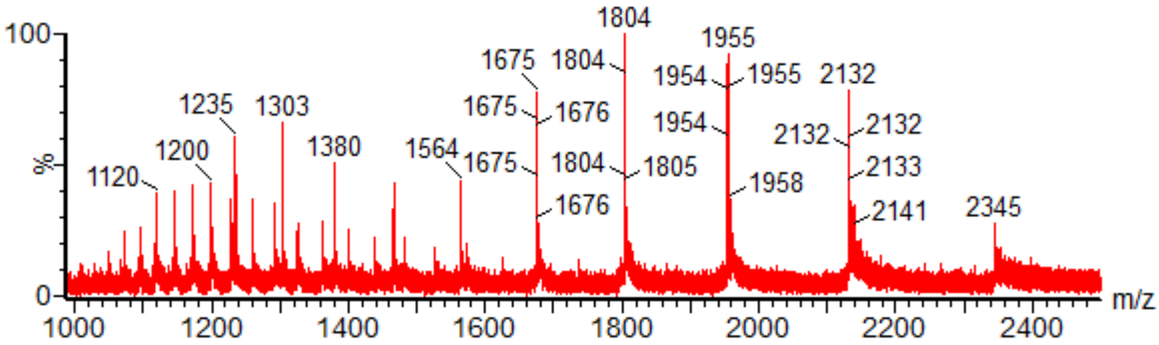

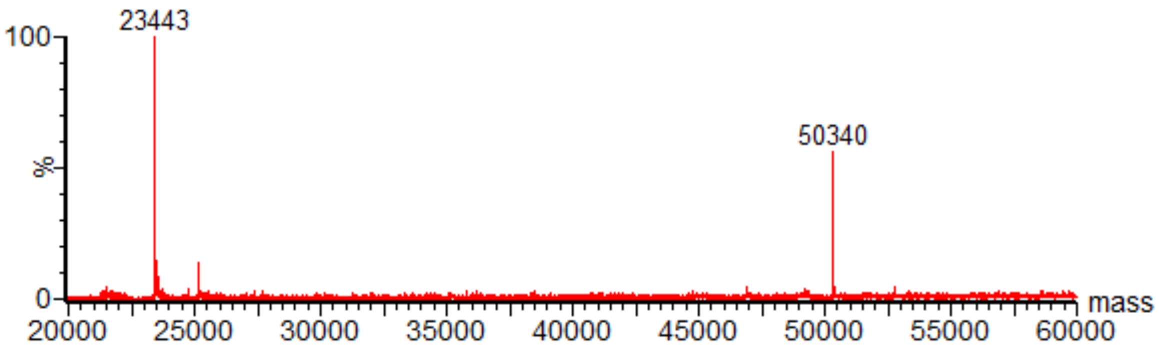

Synthesis of $[DFO]_2$-dg-Her 7

To a solution of azido-dg-Her **4** (400 µL, 1.5 mg/mL in PBS) was added DFO-alkyne **5** (40 µL, 10 eq., 1 mM in DMSO) and the reaction mixture was left at 37 °C for 4 h, with shaking (250 rpm). After confirming by LCMS under reducing conditions that the reaction reached completion, the antibody $[DFO]_2$-dg-Her **7** was purified using 50 MW kDa centrifugal filters by repeated washes with PBS.

Light chain: Theoretical mass 23443 Da; Observed mass: 23436 Da

Heavy chain: Theoretical mass 50233 Da; Observed mass: 50250 Da

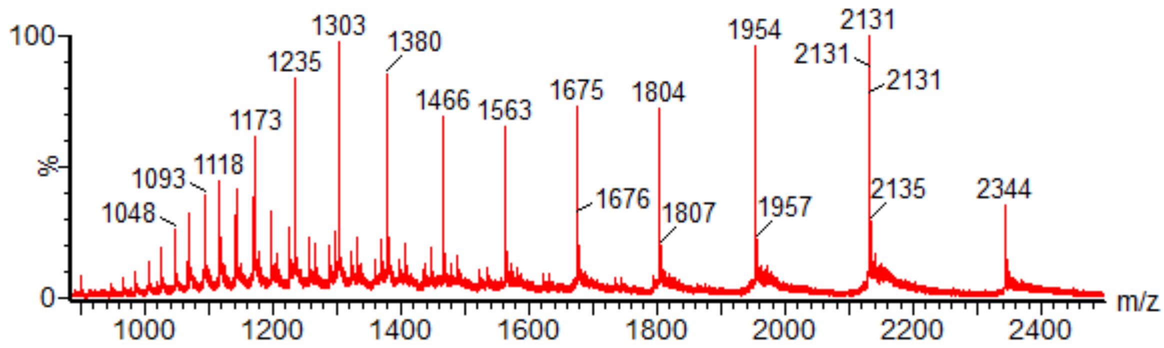

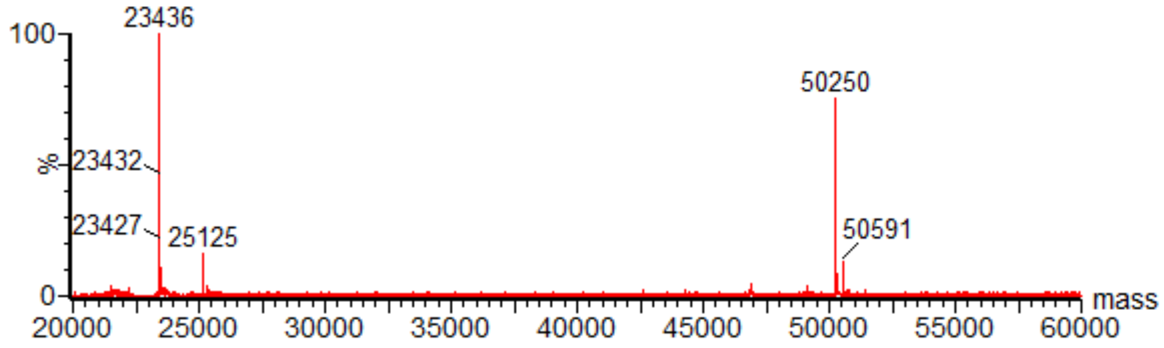

Synthesis of $[DFO+IL1\alpha]_2$-dg-Her 8

To a solution of azido-dg-Her **4** (232 µL, 3 mg/mL in PBS) was added IL1α+DFO-alkyne **5** (46.7 µL, 10 eq. 1 mM in PBS) and the reaction mixture was left at 37 °C for 16 h, with shaking (250 rpm). After confirming by SDS-PAGE and LCMS under reducing conditions that the reaction reached completion, $[DFO+IL1\alpha]_2$-dg-Her **8** antibody conjugate was purified using 50 MW kDa centrifugal filters by repeated washes with PBS.

Light chain: Theoretical mass 23443 Da; Observed mass: 23436 Da

Heavy chain: Theoretical mass 51543 Da; Observed mass: 51552 Da

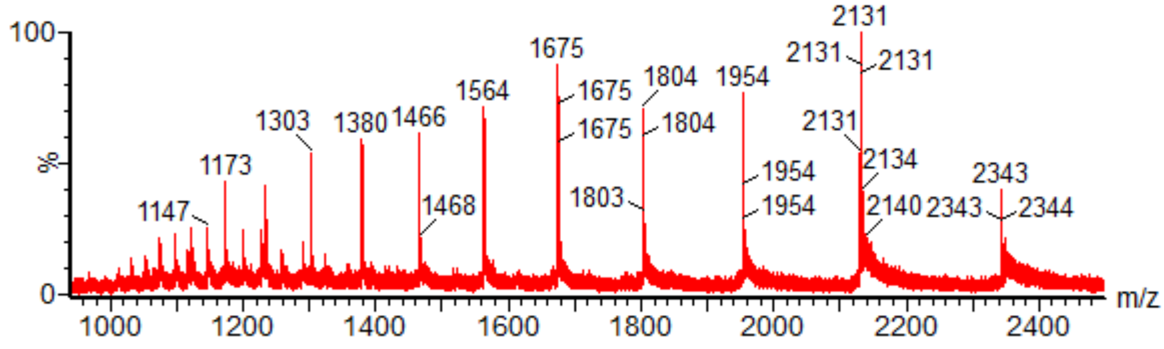

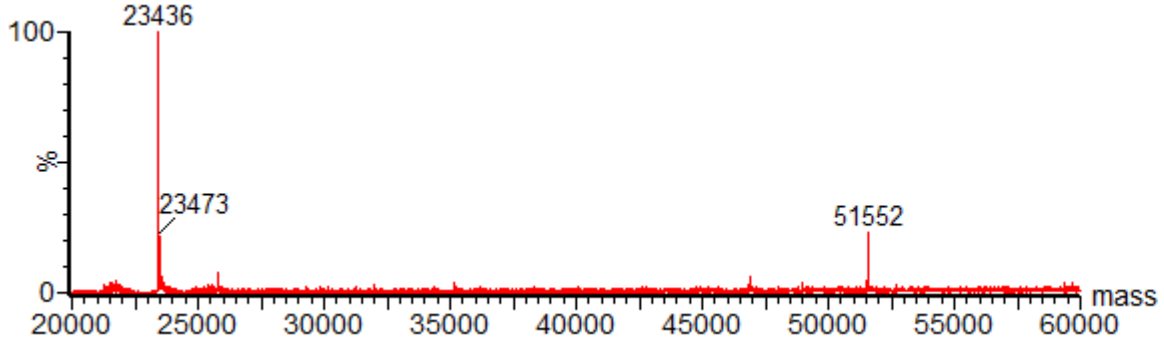

Synthesis of randomly modified [DFO]$_{mix}$-Her 11[3]

To a solution of wt-Her **1** (167 µL, 6 mg/mL in 0.1 M $NaHCO_3$ pH 8.9) was added DFO-Bn-*p*NCS (6.67 µL, 10 eq., 10 mM stock solution in DMSO) and the reaction was left at 37 °C for 1 h, with shaking (250 rpm). After this time, [DFO]$_{mix}$-Her **11** antibody conjugate was purified using 50 MW kDa centrifugal filters by repeated washes with PBS.

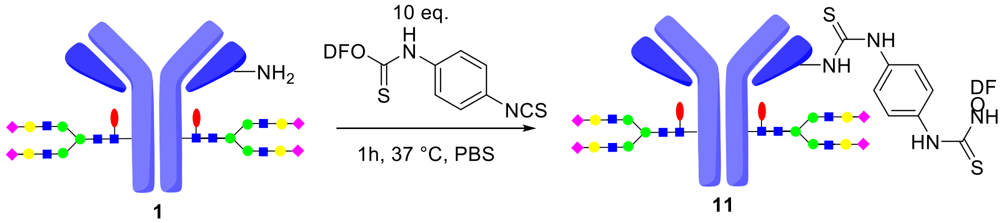

1 **11**

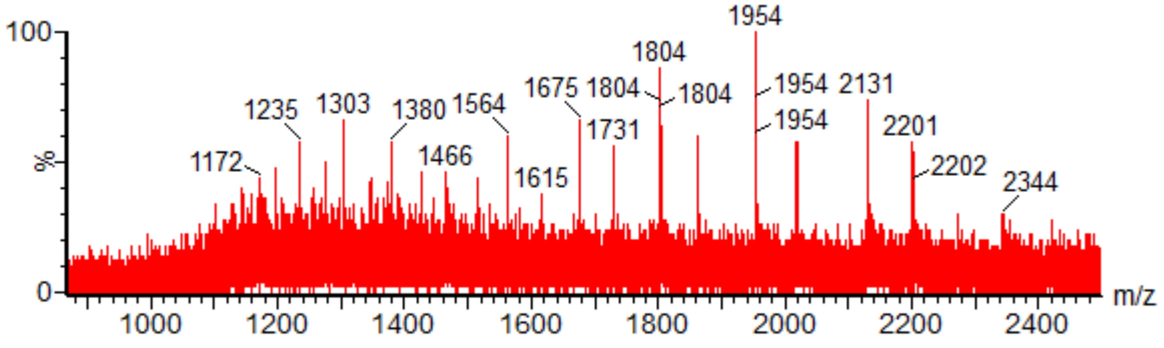

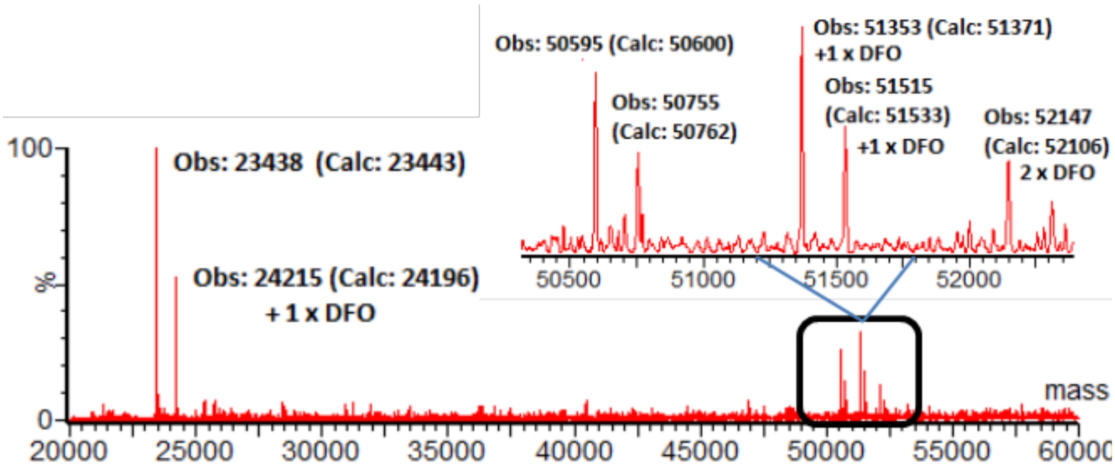

7.3 SDS-PAGE

Aliquots (2 μg of antibody) were treated with reducing loading buffer (NuPAGE® LDS Sample Buffer (4X), supplemented with 400 mM 2-mercaptoethanol) and topped up with water to obtain a final volume of 10 μL. The samples were incubated at 90 °C for 5 min, spun down and loaded onto a 4-12 % Bis-Tris gel. The gel was run at a constant voltage 200 V for 1 h -1h 30 min.

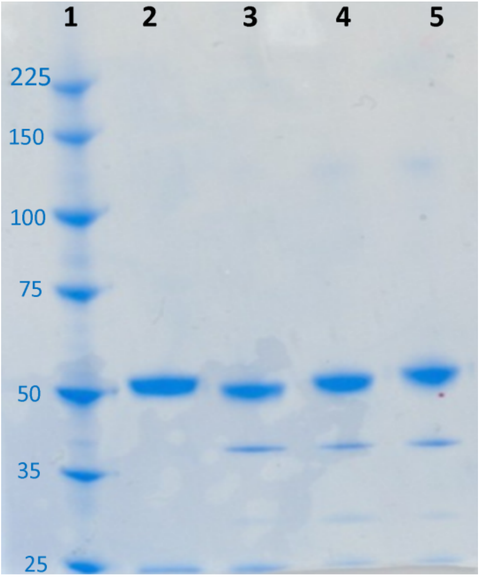

1- Protein ladder (10- 225 kDa)

2- wt-Her **1** (heavy chain)

3- azido-dg-Her **4** (heavy chain)

4- $[DFO]_2$-dg-Her **7** (heavy chain)

5- $[DFO+IL1\alpha]_2$-dg-Her **8** (heavy chain)

Note: the faint band above between 35- 50 kDa represents residual TGase from the azide incorporation reaction.

Attempted transamidation using reduced equivalents of 3 (Method E).

To a solution of deglycosylated Herceptin **2** in PBS were added polyetherazidoamine **3** (40 equivalents) and TGase enzyme (6 U/mg antibody). The reaction was incubated at 37 °C with shaking (250 rpm for 20 h. After this time, the samples were passed through a 50kDa filter membrane and washed with PBS to remove unreacted azide and the TGase enzyme and analysed by rLCMS.

Light chain: Theoretical mass 23443 Da; Observed mass: 23444 Da

Heavy chain: Theoretical mass 49357 Da; Observed mass: 49356 Da

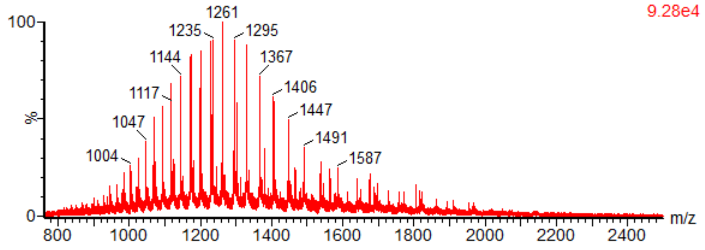

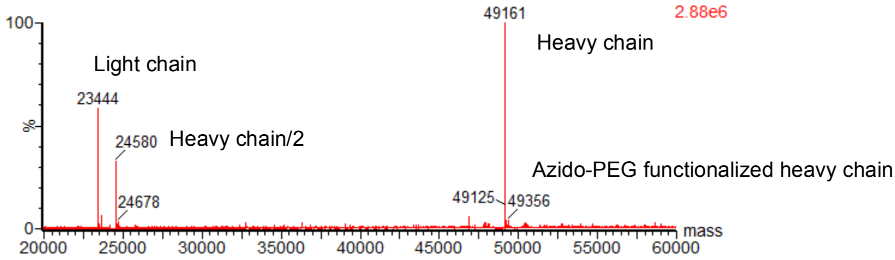

The LCMS indicated the expected product was formed to an extent of ~5%. Decreasing the number of equivalents of azide from 80 equivalents to 40 equivalents resulted in a higher percentage of non-functionalized dgHer-2. This indicated that the reaction required a higher equivalents of azide to push it to completion.

8. Native Mass Spectrometry Methods

8. 1 Sample Preparation for native mass spectrometry experiments

Samples of antibodies were dialysed overnight against 50 mM ammonium bicarbonate the concentration adjusted to 0.5 mg/mL. Additional desalting was sometimes performed by further buffer exchange into freshly prepared ammonium bicarbonate 50 mM using P6 Biospin columns (Bio-Rad) or Zeba Spin desalting columns (7K or 40K) (Thermo Fisher). Concentration was reduced as necessary for nMS.

8.2 High Resolution Native Mass Spectrometry

High resolution native mass spectrometry was performed as described elsewhere.[2] Briefly, ions were introduced into a Q Exactive hybrid quadrupole-Orbitrap (Thermo Fisher, Bremen Germany) mass spectrometer modified for the transmission and detection of high *mass* ions.[4,5] All spectra were acquired in "Native Mode" with maximum RF applied to all ion optics, -3.2 kV to the central electrode of the Orbitrap and with ion trapping in the HCD cell. Ions were generated in the positive ion mode from a static nanospray source (+1.0 to +1.4 kV) using gold-coated capillaries prepared in-house.[6] Ions then passed through a temperature controlled transfer tube (40-80 ^{o}C), RF-lens, injection flatapole and bent flatapole. After traversing the selection quadrupole, which was operated with a wide selection window (2,000-15,000 *m/z*), ions were trapped in the HCD cell before being transferred into the C-trap and Orbitrap mass analyser for detection. Transient times were 64 ms and AGC target was 1×10^{6}. Spectra were acquired with either 1 or 10 microscans, averaged over 50-100 scans and with a noise level parameter set to 3, slightly lower than the default of 4.68. Efficient desolvation of intact antibodies was achieved through increased voltages applied in the HCD cell (150-200 V). No in-source activation was applied. The collision gas was Argon and pressure in the HCD cell was maintained to achieve a UHV pressure of approximately 1×10^{-9} mbar. Data was processed using Thermo Scientific Xcalibur 2.1 and masses and S.D. calculated using in-house software (http://benesch.chem.ox.ac.uk/resources.html) using the three most abundant charge states. The reaction product abundances were calculated (where possible) based on the peak intensities on the three most abundant charge states as described previously[2].

When performing nMS analysis on modified antibodies conditions were kept as "gentle" as possible to avoid dissociation of conjugated moieties as previously described[2]. The resulting peaks were often slightly asymmetric (with a tail to the right hand side) and peak widths were consistently around twice that expected purely from the isotopic distribution. This peak broadening is likely due to complexation with salts and other small molecule adducts that have not been completely removed during desolvation.[7] This

incomplete desolvation likely explains the consistently higher measured masses we report compared to the theoretical mass. It may also be responsible to the slightly elevated standard deviation in measured mass (as adduct retention is likely to be charge state dependant, and thus increase S.D.). In the case of highly conjugated antibody constructs such as **7** and more so with **8**, desolvation was particularly challenging. This is likely due to the presence of the DFO moiety which may strongly chelate various metal ions such as Fe. Methods to achieve more exhaustive salt removal remain the subject of ongoing work.

8.3 Native mass spectra of azido-dg-Her 4

azido-dg-Her 4 sample obtained using method A

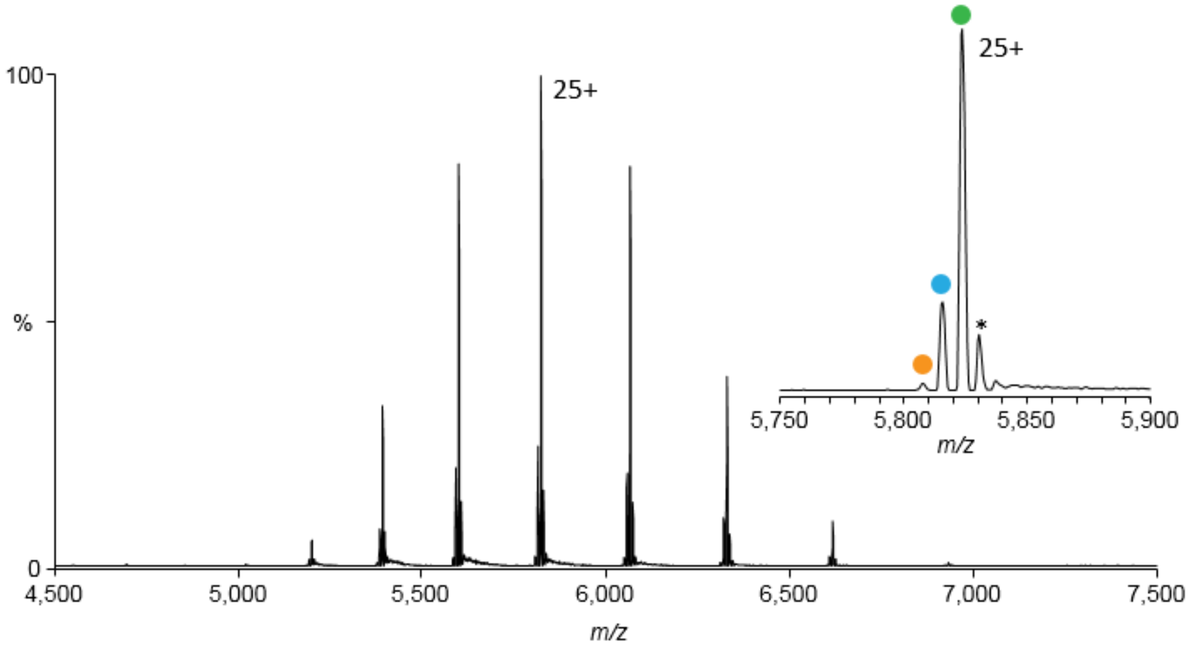

	dg-Her 2	$[N_3]$-dg-Her 4a	$[N_3]_2$-dg-Her 4b	$[N_3]_2$-*
**M/26**	5584.57	5592.22	5599.82	5606.43
**M/25**	5807.95	5815.79	5823.73	5830.63
**M/24**	6049.93	6058.00	6066.39	6073.64
**M.W. observed (Da)**	145173	145369	145569	145741
**M.W. S.D.**	1	2	1	1
**M.W. theoretical (Da)**	145167	145368	145569	145745
**Abundance (%)**	1.6	16.7	70.2	11.5
**S.D. Abundance (%)**	0.2	0.3	0.6	0.4

azido-dg-Her 4 sample obtained using Method B

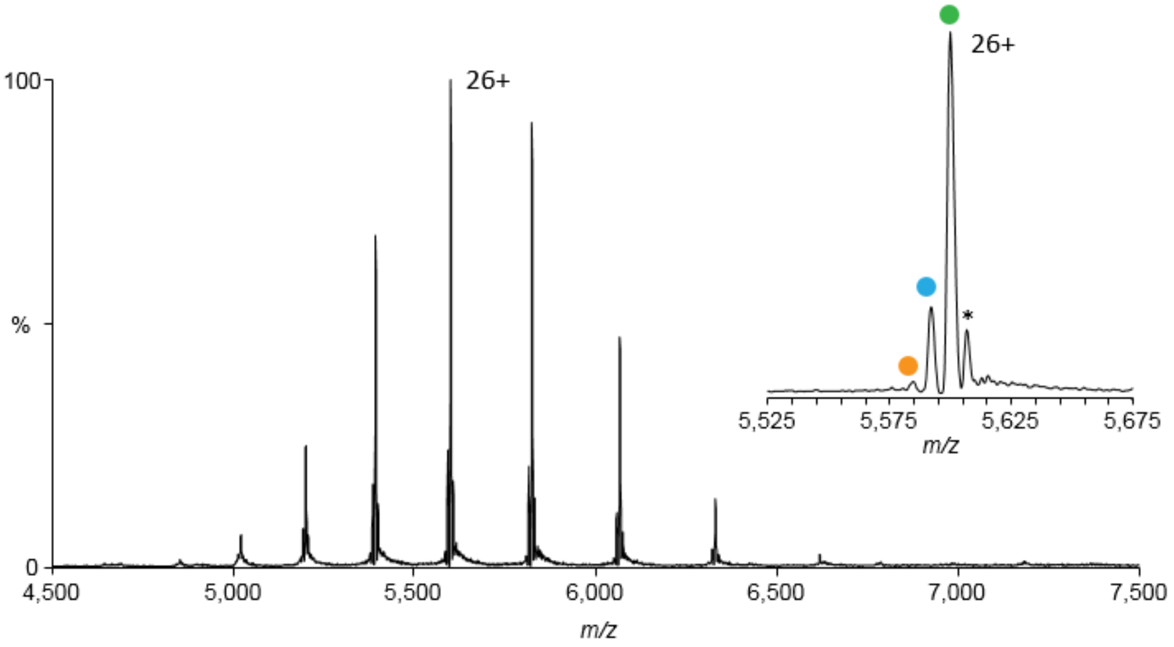

	dg-Her 2	[N_3]-dg-Her 4a	[N_3]$_2$-dg-Her 4b	[N_3]$_2$-*
**M/27**	5377.56	5385.33	5392.62	5399.87
**M/26**	5584.88	5592.25	5600.11	5606.89
**M/25**	5807.67	5815.74	5824.14	5831.58
**M.W. observed (Da)**	145171	145372	145576	145762
**M.W. S.D.**	8	4	2	8
**M.W. theoretical (Da)**	145167	145368	145569	145745
**Abundance (%)**	2.5	16.2	69.7	11.5
**S.D. Abundance (%)**	0.8	0.9	1.8	0.8

azido-dg-Her 4 sample obtained using Method C

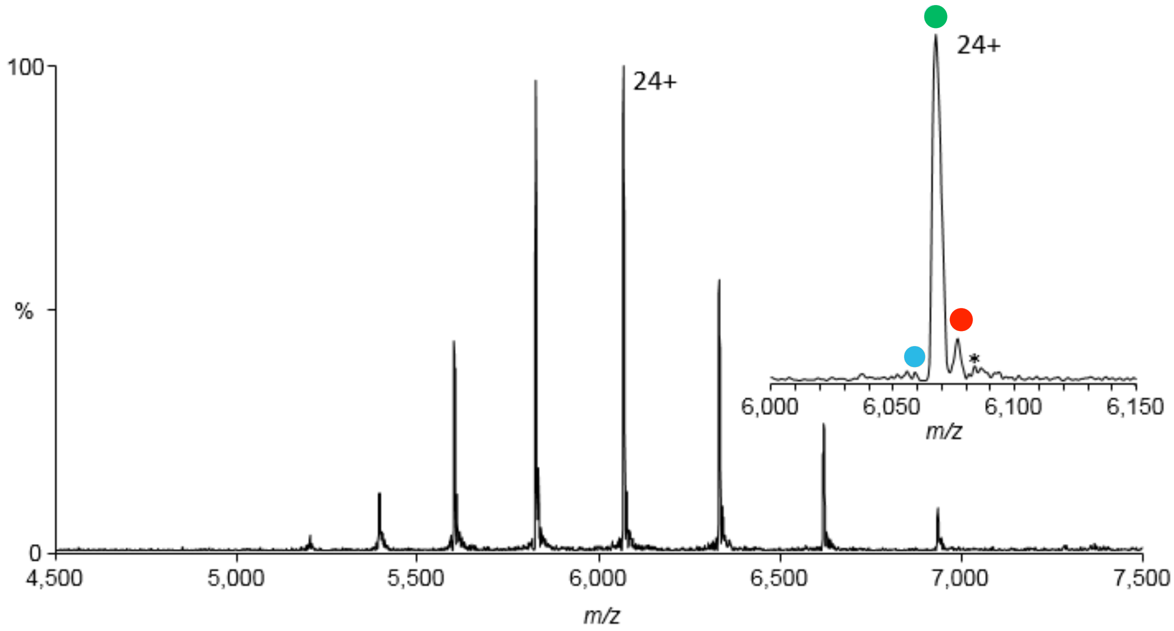

	$[N_3]$-dg-Her 4a	$[N_3]_2$-dg-Her 4b	$[N_3]_3$-dg-Her 4c	$[N_3]_3$- *
**M/26**	5816.16	5825.29	5833.29	5840.66
**M/24**	6059.29	6068.05	6076.35	6083.66
**M/25**	6322.61	6331.68	6341.14	6347.78
**M.W. observed (Da)**	145392	145607	145812	145984
**M.W. S.D.**	11	2	9	8
**M.W. theoretical (Da)**	145368	145569	145770	145946
**Abundance (%)**	2.4	80.4	14.4	2.9
**S.D. Abundance (%)**	0.9	1.8	1.5	0.9

azido-dg-Her 4 sample obtained using Method D

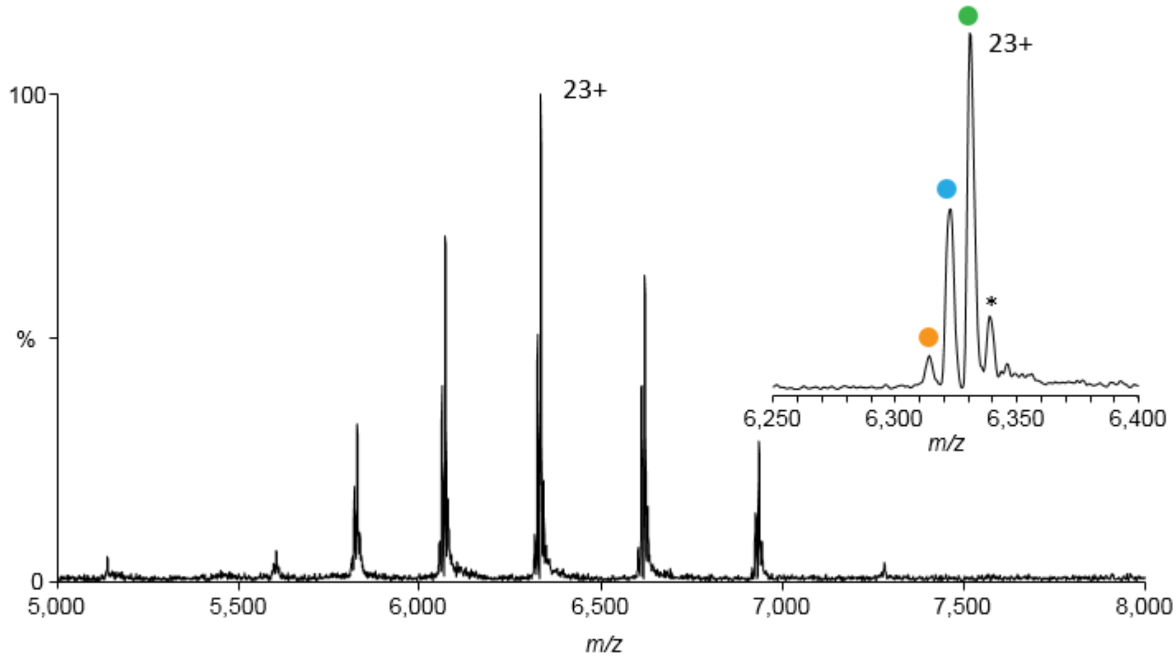

	dg-Her 2 (orange)	$[N_3]$-dg-Her 4a (blue)	$[N_3]_2$-dg-Her 4b (green)	$[N_3]_2$-*
**M/24**	6051.48	6058.82	6067.59	6074.33
**M/23**	6313.17	6322.87	6331.00	6339.05
**M/22**	6600.70	6609.93	6618.59	6626.87
**M.W. observed (Da)**	145195	145396	145591	145768
**M.W. S.D.**	16	8	6	8
**M.W. theoretical (Da)**	145167	145368	145569	145946
**Abundance (%)**	4.9	29.8	53.6	11.7
**S.D. Abundance (%)**	1.4	1.6	1.2	0.7

8.4. Native mass spectra of anti-γH2Ax

nMS for anti-γH2AX trimmed with PNGase

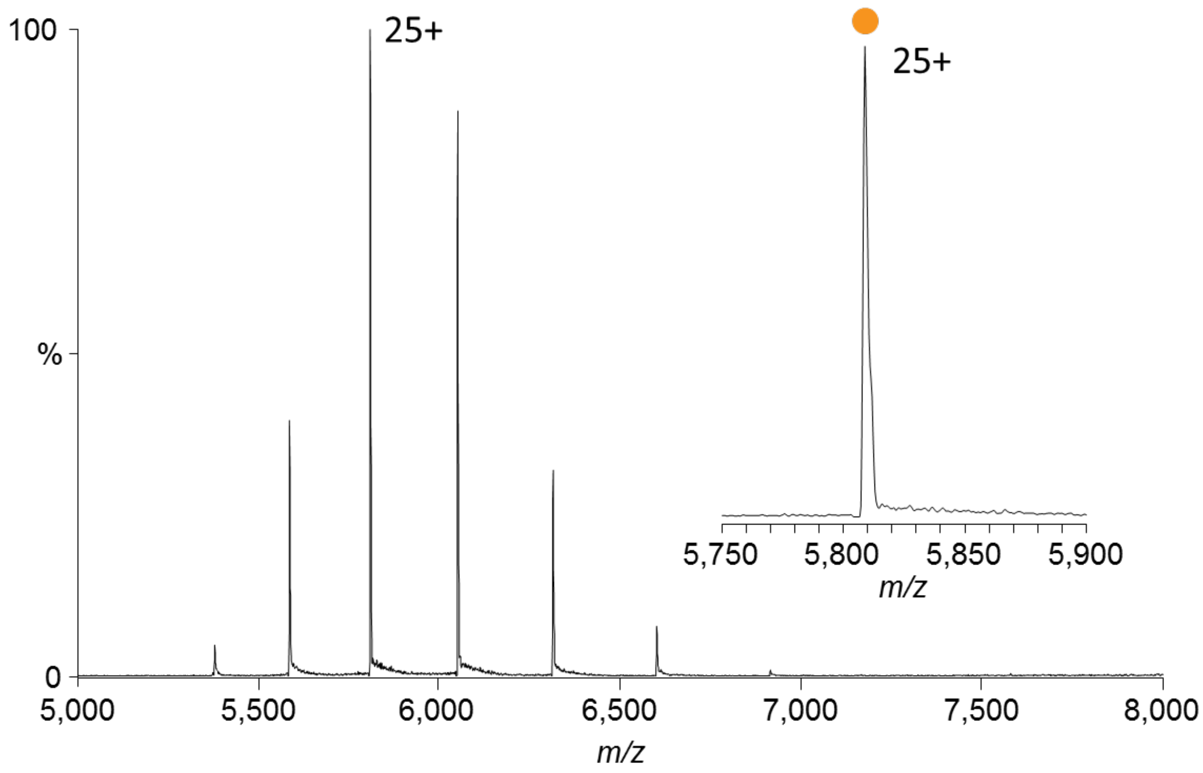

nMS data for reaction A

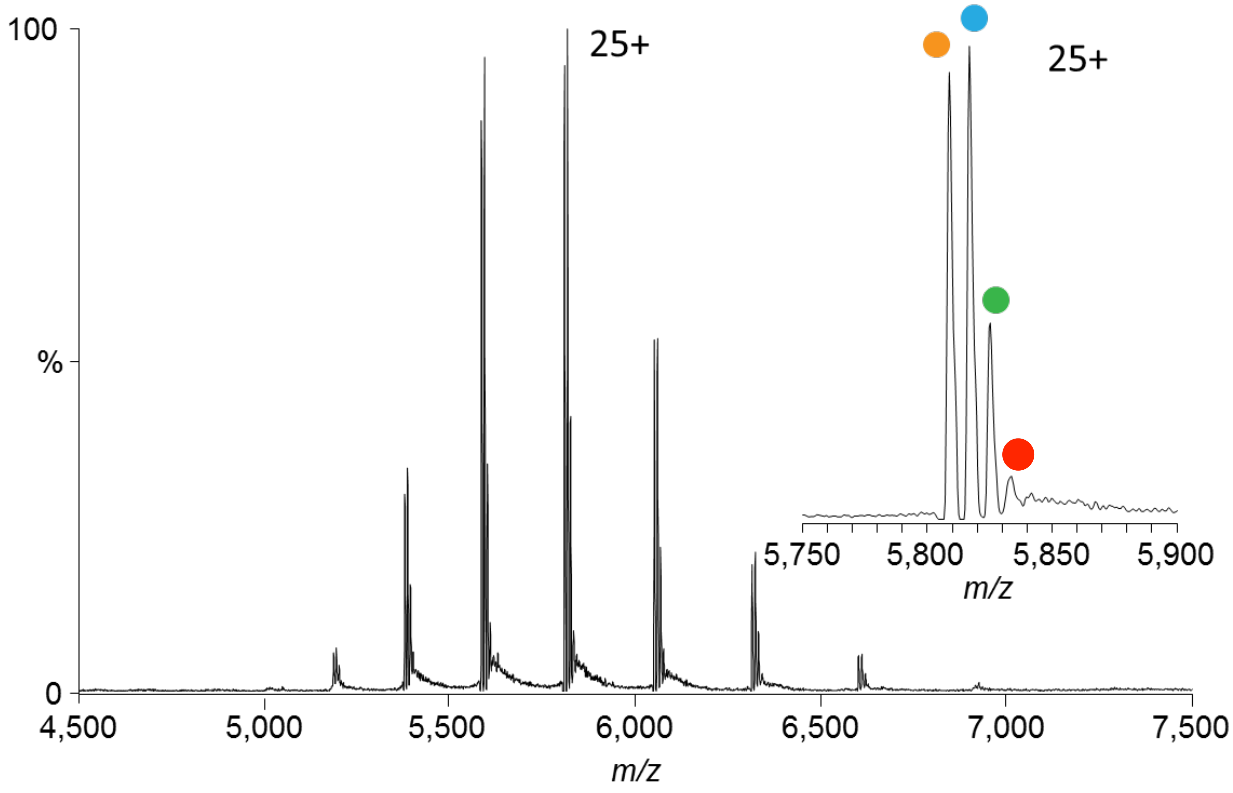

nMS data for reaction B

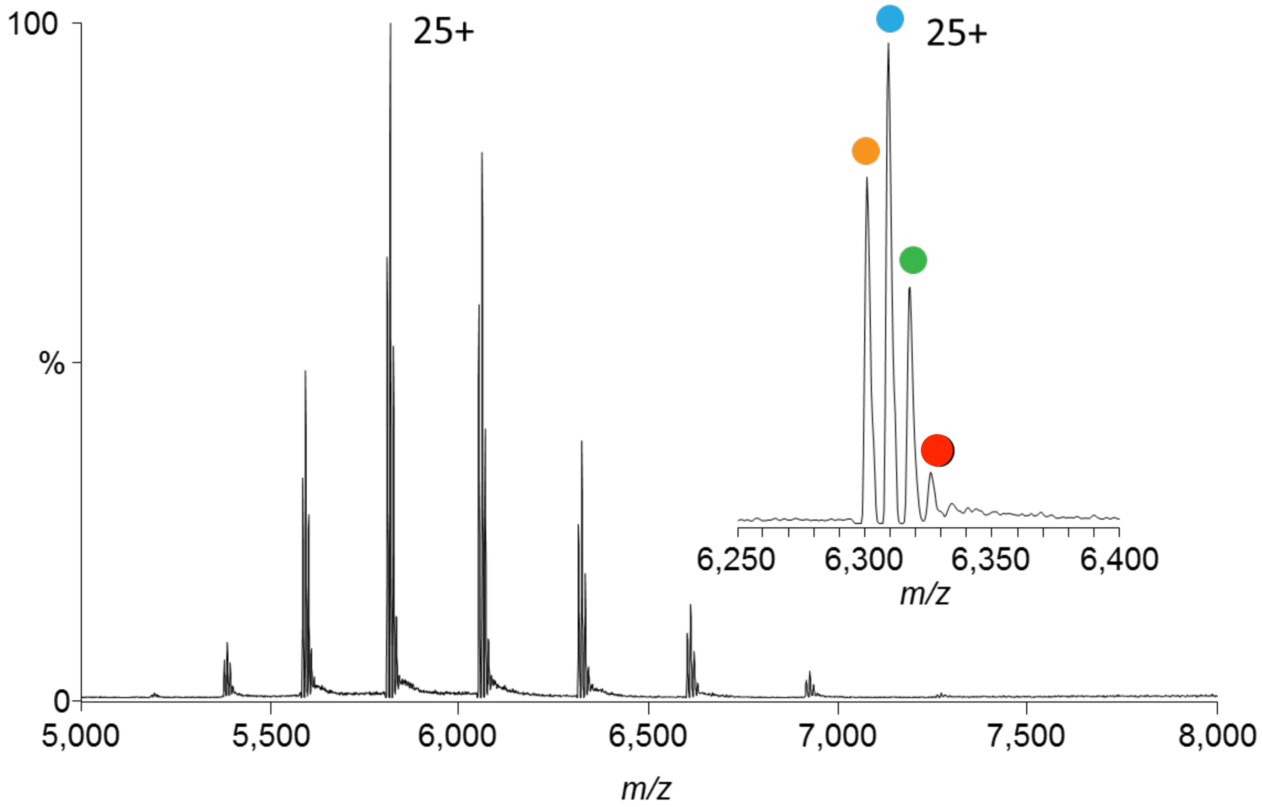

nMS data for reaction C

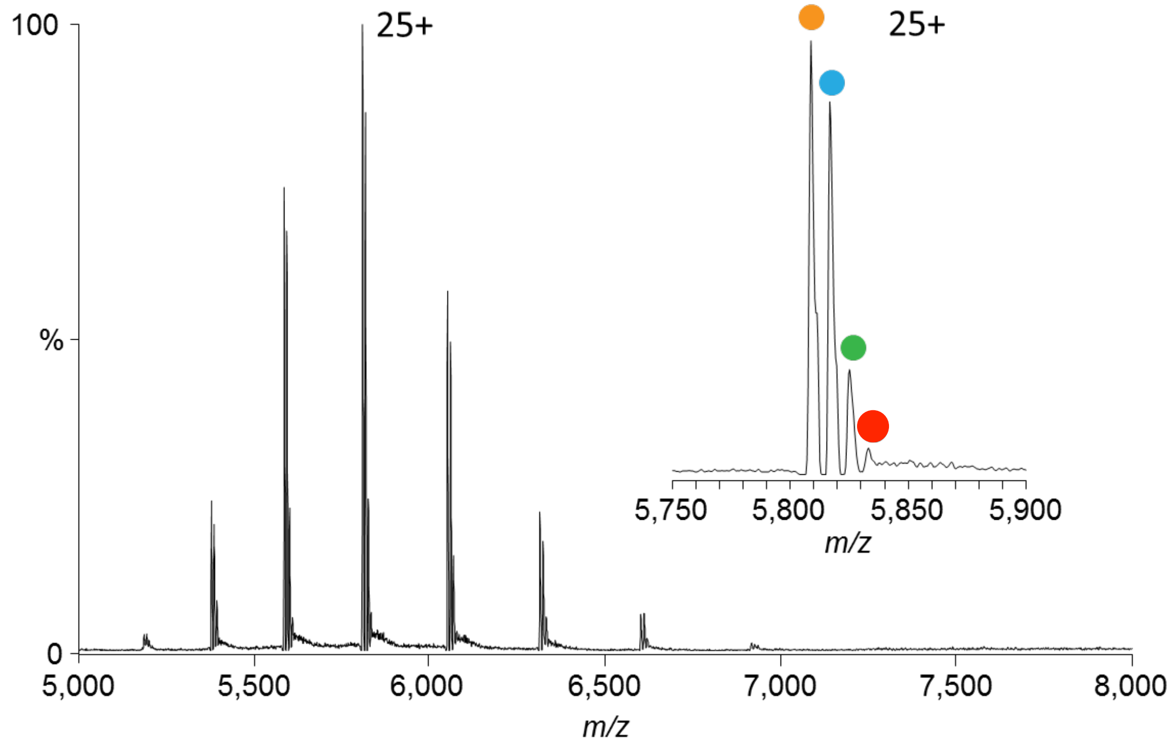

nMS data for reaction D

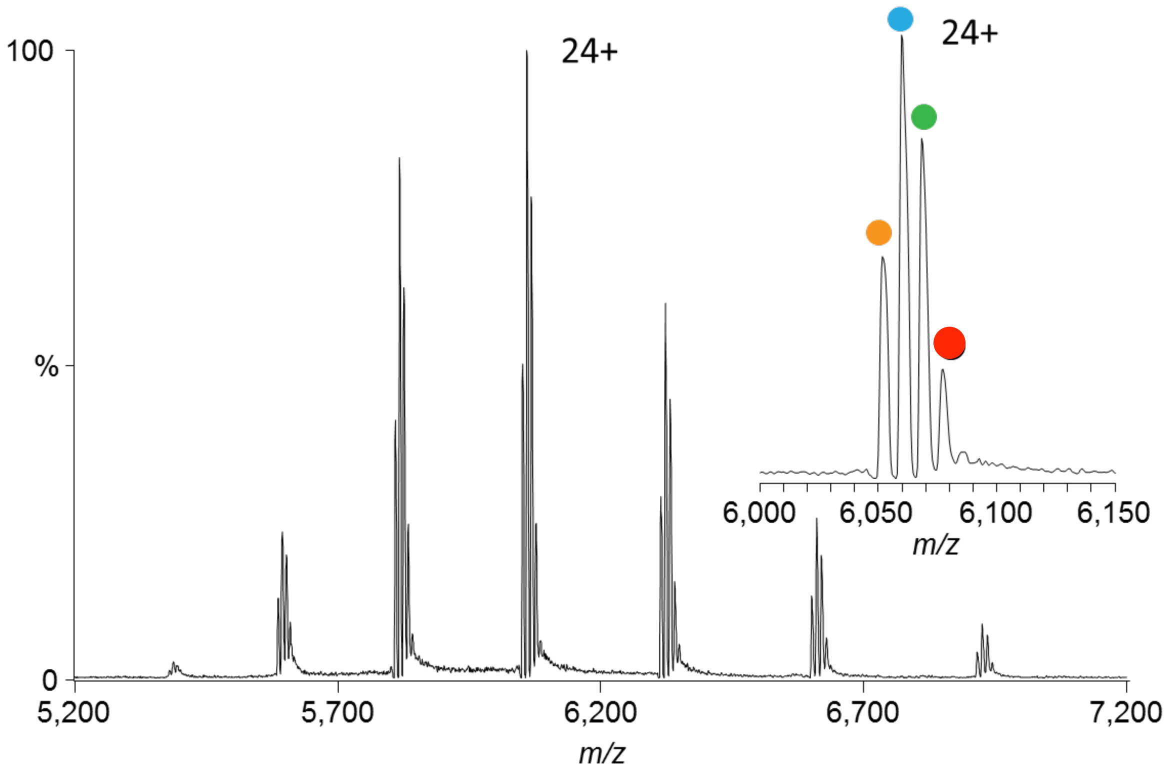

9. MS/MS Methods

9.1 Digest of azido-dg-Her 4 sample for MS/MS analysis

azido-dg-Her **4** (30 µg, 7.2 mg/mL) was diluted with 8 M urea, 50 mM ammonium biocarbonate buffer to give a final concentration of 0.1 mg/mL of **3**. To this solution, DTT (180 µg, 30 mg/mL in water) was added and the resulting mixture was incubated at 56 °C for 25 min. After this time, iodoacetamide (156 µg, 26 mg/mL in 8 M urea, 50 mM ammonium bicarbonate buffer) was added and the resulting mixture was incubated at room temperature, for 30 min, in the dark. At the end of this incubation step, another portion of DTT (180 µg, 30 mg/mL in water) was added, followed by 600 µL of 50 mM ammonium bicarbonate buffer. Finally, trypsin (1.2 µg, 2 mg/mL in water) was added and the reaction mixture was incubated at 37 °C overnight.

9.2 MS/MS analysis method

Peptides were re-suspended in 10% formic acid. They were separated on an Ultimate 3000 UHPLC system (Thermo Fischer Scientific) and electrosprayed directly into a QExactive mass spectrometer (Thermo Fischer Scientific) through an EASY-Spray nano-electrospray ion source (Thermo Fischer Scientific). The peptides

were trapped on a C18 PepMap100 pre-column (300 µm i.d. x 5 mm, 100Å, Thermo Fisher Scientific) using solvent A (0.1% Formic Acid in water) at a pressure of 500 bar. The peptides were separated on an in-house packed analytical column (75 µm i.d. packed with ReproSil-Pur 120 C18-AQ, 1.9 µm, 120 Å, Dr.Maisch GmbH) using a linear gradient (length: 120 minutes, 7% to 28% solvent B (0.1% formic acid in acetonitrile), flow rate: 200 nL/min). The raw data was acquired on the mass spectrometer in a data-dependent mode (DDA). Full scan MS spectra were acquired in the Orbitrap (scan range 350-2000 m/z, resolution 70000, AGC target 3e6, maximum injection time 50 ms). After the MS scans, the 20 most intense peaks were selected for HCD fragmentation at 30% of normalised collision energy. HCD spectra were also acquired in the Orbitrap (resolution 17500, AGC target 5e4, maximum injection time 120 ms) with first fixed mass at 180 m/z.

10. Antibody Radioloading Methods

An aliquot corresponding to 10 MBq of ^{89}Zirconium 1M in oxalic acid was adjusted to pH 7 - 8 by addition of 1 M Na_2CO_3. The resulting solution was added to the DFO-labelled antibody constructs **7**,8 and **11** respectively (100 µg, 2 mg/mL) and the mixture was incubated at room temperature for 1 h. The radiolabelling efficiency was assessed by iTLC using an eluent of 50 mM EDTA 9 pH 5.8. The radiolabelled antibodies were purified on a G50 size-exclusion column, eluting with PBS.

10.1 Stability of [^{89}Zr·DFO+IL1α]$_2$-dg-DFO 10 in human serum

An aliquot of [^{89}Zr·DFO+IL1α]$_2$-dg-Her **10** (10 µL, 0.15 MBq) was mixed with 40 µL human serum. The experiment was performed in triplicate. The resulting solutions were incubated at 37° C and analysed by iTLC over 96 h. The rest of the purified antibody (200 µL, 2.85 MBq) was stored in PBS, at 4 ° C. iTLC analysis of the stock solution was performed at various time points over 96 h.

11. *In vitro* binding assay

MDA-MB-231/H2N cells (3x10^4 cells per well)[8] were seeded in a 96-well plate and left to adhere overnight. After this time, the growth medium was removed and the wells were washed with wash buffer (0.05% Tween in PBS, 3 x 5 min). Blocking was achieved using 2% BSA in PBS for 1 hour at room temperature. Following washing with wash buffer (1 x 5 min), wt-Her **1** and conjugates **7**, **8** and **11** were applied to the wells, in a 3x serial dilution, covering a 0-405 nM concentration range. After 1 h incubation at 4 °C, the wells were washed -with wash buffer (3 x 5 min), and the secondary antibody, HRP-conjugated rabbit anti human IgG (Invitrogen, #656120) was applied (1 : 20000 dilution in blocking buffer). After 1 h of incubation at room temperature, the wells were washed with wash buffer (6 x 5 min). The wash buffer was removed and the

TMB developing substrate (100 µL, Thermo Scientific) was added. The reaction was stopped, after 30 min at room temperature, with 1M sulfuric acid (100 µL). The absorbance at 450 nm was then determined using a plate reader. The absorbance at 450 nm was plotted against the concentration of the primary antibody (Figure **S4**). Experimental data was fitted using a non-linear one site specific binding algorithm (Graph Pad Prism), constraining the background and the non-specific binding to zero.

12. Abbreviations

S.D. = standard deviation

rLCMS = Liquid chromatography couples to mass spectrometry under reducing conditions

mAb= monoclonal antibody

dg=deglycosylated

13. References

(1) Nebija, D.; Kopelent-Frank, H.; Urban, E.; Noe, C. R.; Lachmann, B. *Journal of Pharmaceutical and Biomedical Analysis* **2011**, *56*, 684.

(2) Parsons, T. B.; Struwe, W. B.; Gault, J.; Yamamoto, K.; Taylor, T. A.; Raj, R.; Wals, K.; Mohammed, S.; Robinson, C. V.; Benesch, J. L. P.; Davis, B. G. *Angewandte Chemie International Edition* **2016**, *55*, 2361.

(3) Knight, J. C.; Paisey, S. J.; Dabkowski, A. M.; Marculescu, C.; Williams, A. S.; Marshall, C.; Cornelissen, B. *Dalton Trans.* **2016**, *45*, 6343.

(4) Gault, J.; Donlan, J. A. C.; Liko, I.; Hopper, J. T. S.; Gupta, K.; Housden, N. G.; Struwe, W. B.; Marty, M. T.; Mize, T.; Bechara, C.; Zhu, Y.; Wu, B.; Kleanthous, C.; Belov, M.; Damoc, E.; Makarov, A.; Robinson, C. V. *Nature methods* **2016**, *13*, 333.

(5) Rose, R. J.; Damoc, E.; Denisov, E.; Makarov, A.; Heck, A. J. R. *Nature methods* **2012**, *9*, 1084.

(6) Hernandez, H.; Robinson, C. V. *Nat. Protoc.* **2007**, *2*, 715.

(7) Lössl, P.; Snijder, J.; Heck, A. J. R. *Journal of The American Society for Mass Spectrometry* **2014**, *25*, 906.

(8) Costantini, D. L.; Bateman, K.; McLarty, K.; Vallis, K. A.; Reilly, R. M. *Journal of Nuclear Medicine* **2008**, *49*, 1498.
